# A body–brain circuit that regulates body inflammatory responses

**DOI:** 10.1038/s41586-024-07469-y

**Published:** 2024-05-01

**Authors:** Hao Jin, Mengtong Li, Eric Jeong, Felipe Castro-Martinez, Charles S. Zuker

**Affiliations:** 1 Zuckerman Mind Brain Behavior Institute, Howard Hughes Medical Institute, Columbia University, New York, NY USA; 2 Department of Neuroscience, Vagelos College of Physicians and Surgeons, Columbia University, New York, NY USA; 3 Department of Biochemistry and Molecular Biophysics, Columbia University, New York, NY USA; 4 Center for Cancer Research, National Cancer Institute, Bethesda, MD USA; 5 Present Address: Laboratory of Host Immunity and Microbiome, National Institute of Allergy and Infectious Diseases, Bethesda, MD USA

**Keywords:** Neural circuits, Molecular neuroscience

## Abstract

The body–brain axis is emerging as a principal conductor of organismal physiology. It senses and controls organ function^1,2^, metabolism^3^ and nutritional state^4–6^. Here we show that a peripheral immune insult strongly activates the body–brain axis to regulate immune responses. We demonstrate that pro-inflammatory and anti-inflammatory cytokines communicate with distinct populations of vagal neurons to inform the brain of an emerging inflammatory response. In turn, the brain tightly modulates the course of the peripheral immune response. Genetic silencing of this body–brain circuit produced unregulated and out-of-control inflammatory responses. By contrast, activating, rather than silencing, this circuit affords neural control of immune responses. We used single-cell RNA sequencing, combined with functional imaging, to identify the circuit components of this neuroimmune axis, and showed that its selective manipulation can effectively suppress the pro-inflammatory response while enhancing an anti-inflammatory state. The brain-evoked transformation of the course of an immune response offers new possibilities in the modulation of a wide range of immune disorders, from autoimmune diseases to cytokine storm and shock.

## Abstract (web summary)

The body–brain axis regulates body pro-inflammatory and anti-inflammatory immune responses following an immune insult.

## Main

A well-balanced immune response is of fundamental importance for the fitness and survival of the organism. An overactive pro-inflammatory state invariably leads to immune dysregulation, including a diverse range of autoimmune and inflammatory diseases^7,8^. Understanding the mechanisms that tune the immune response may afford important insights into the function of the immune system, and provide novel strategies to combat disorders and diseases characterized by dysregulated immune states.

Much is known about innate^9^ and adaptive immunity^10^, with numerous cellular and humoral factors having essential roles in initiating, amplifying and terminating immune responses^11–13^. Several studies have shown that infection can activate neural circuits mediating physiologically conserved responses such as fever, malaise and changes in feeding behaviour^14–17^, and pioneering work by Tracey and collaborators have revealed the significance of electrical stimulation of the vagal nerve as a therapeutic strategy to attenuate inflammation^18^. However, how the brain, as the central ‘arbiter’ of body physiology, regulates immunity remains poorly understood, despite our knowledge of several potential pathways linking the brain to immune cells^19–23^.

Here we describe a body–brain neural circuit that informs the brain of an emerging inflammatory response. We identified vagal neurons that respond to pro-inflammatory versus anti-inflammatory immune mediators, and showed that they signal to a genetically defined population of neurons in the brainstem to modulate and shape the course on an inflammatory response. These results reveal the influence of the body–brain axis in controlling innate immunity and highlight the therapeutic potential of recruiting this axis to help rebalance immune function.

## Neurons activated by innate immunity

The brain monitors nearly all aspects of body biology, including responses to infection^14^, internal-state changes^24^, sickness and inflammation^15,16,25,26^. The notion that the brain and immune systems interact with each other has long been proposed^21,27^. However, the identity of the circuit elements linking peripheral immunity with the brain have remained largely unknown. We reasoned that if we could identify neuronal populations in the brain that are activated by a peripheral immune insult, it would help to dissect the neural control of immunity.

**Fig. 1:**
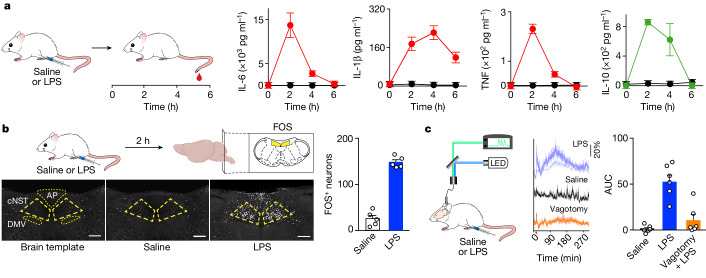
Immune responses activate the brain via the vagal–brain axis. **a**, Schematic illustrating LPS-induced cytokine measurements (left). Wild-type mice were injected with saline or LPS, and peripheral blood was sampled every 2 h. Also shown are levels of IL-6, IL-1β, TNF and IL-10 by ELISA. LPS is in red or green; saline is in black; *n* = 5 mice. Values are means ± s.e.m. **b**, Schematic of FOS induction by LPS stimulation (top). Mice received an intraperitoneal injection of saline or LPS. Two hours later, brains were immunostained for FOS expression. Strong bilateral FOS labelling is detected in neurons of the cNST (highlighted in yellow) in LPS-stimulated mice (bottom); *n* = 5 mice. Quantification of FOS-positive neurons; the equivalent area of the cNST (200 × 200 μm, Bregma −7.5 mm) was processed for each sample (right). Values are mean ± s.e.m; Mann–Whitney *U*-tests, *P* = 0.008. Scale bars, 200 μm. AP, area postrema; DMV, dorsal motor vagal complex. **c**, Fibre photometry of LPS-evoked activity in the cNST (left). A GCaMP6s AAV was targeted to the cNST of *Vglut2-cre* mice (see Extended Data Fig. 5e). Neural responses following LPS (dark blue traces, 0.5 mg kg^−1^, *n* = 6; light blue traces, 0.1 mg kg^−1^, *n* = 4) and control saline (black traces, *n* = 6) (middle). Traces display mean (solid) and s.e.m. (shaded). The orange traces depict responses after bilateral vagotomy (*n* = 6). The saline and LPS injections were done as successive stimulations in the same animals. Scale bar, Δ*F/F*. The red arrow indicates time of injections. Quantification of responses (right). AUC, area under the curve. Values are mean ± s.e.m.; Wilcoxon test (saline versus LPS), *P* = 0.03; Mann–Whitney *U*-test (LPS versus vagotomy), *P* = 0.004; Mann–Whitney *U*-test (saline versus vagotomy), *P* = 0.18. Note the severe loss of LPS-evoked responses (approximately 80%) following removal of the vagal communication pathway. Schematics were created using BioRender (https://biorender.com).

**Extended Data Fig. 1:**
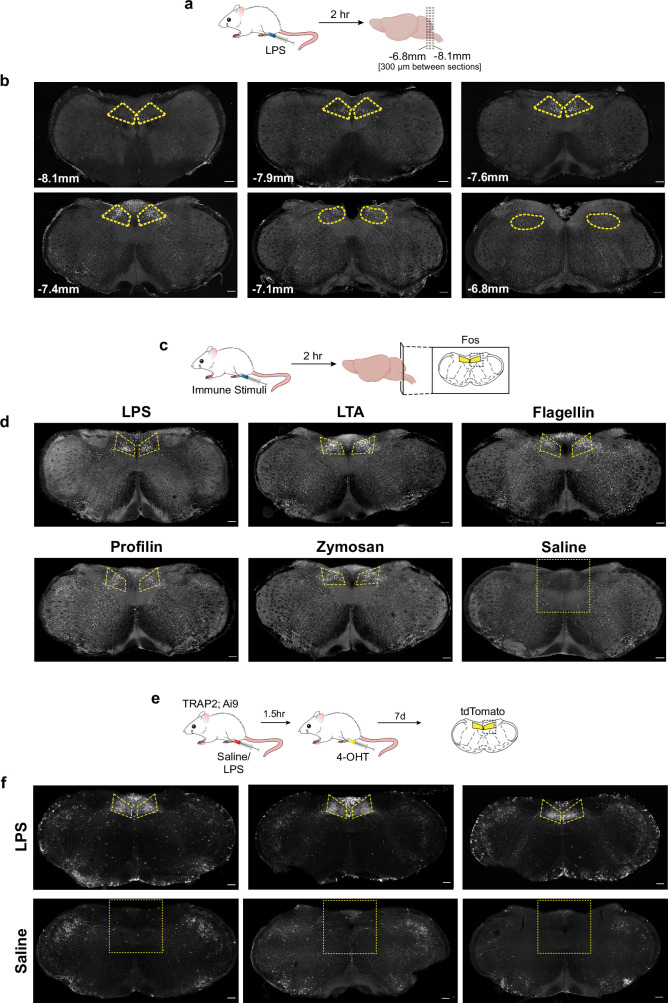
cNST neurons activated in response to immune insults. **a**, Schematic of Fos induction by LPS stimulation. Mice received an intraperitoneal injection with LPS, and two hours later, brains were extracted, sliced and immune-stained for Fos expression. **b**, Shown is Fos expression in six 100 μm coronal sections, each 300 μm apart from bregma −8.1mm to bregma −6.8mm. Note the selective induction of Fos in the cNST but not in the rostral nucleus of the solitary tract (rNST). Scale bars, 200 μm. Similar results were observed in multiple animals (n = 4). **c**, Fos is induced by a variety of immune insults. Schematic of Fos induction by immune stimulation. Mice received an intraperitoneal injection with a variety of different immune challenges, and two hours later, brains were extracted, and immuno-stained for Fos expression. **d**, Shown are examples for LPS (50 μg kg^−1^), Lipoteichoic acid (LTA, 1 mg kg^−1^), Flagellin (20 μg kg^−1^), Profilin (20 μg kg^−1^) and Zymosan (2.5 mg kg^−1^). All robustly activated Fos in the cNST (outlined in yellow). Scale bar, 200 μm. **e**, Schematic illustrating experimental procedures to TRAP cNST neurons activated by LPS. We genetically labelled the LPS-induced TRAPed neurons with a Cre-dependent fluorescent reporter (tdTomato, Ai9^63^). TRAP2;Ai9 mice were stimulated intraperitoneally with LPS (50 μg kg^−1^) or control (saline) stimulus, followed by injection of 4-OHT 90 mins later. After 7 days, the brains were sectioned and examined for the induction of the tdTomato reporter. **f**, Shown are coronal sections of cNST after TRAP2;Ai9 animals were TRAPed with LPS or Saline. Each panel is a confocal maximal projection image from Bregma −7.5 mm. Shown are data representing 3 different animals, in independent experiments. Note that LPS but not saline led to consistent and robust bilateral TRAP labelling of neurons in the cNST(outlined in yellow) across animals. Scale bars, 200 μm. Schematics in panels **a**,**c**,**e** were created using BioRender (https://biorender.com).

We used lipopolysaccahride (LPS), a canonical immune stimulus derived from the outer membrane of Gram-negative bacteria to elicit innate immune responses^28^. We challenged separate cohorts of mice with intraperitoneal injection of LPS and vehicle control (saline), and then examined the evoked immune response by measuring cytokine changes^28,29^ in peripheral blood samples. As expected, a single dose of LPS is sufficient to trigger significant increases in the levels of pro-inflammatory and anti-inflammatory cytokines, with a time course peaking at about 2 h after LPS injection (Fig. 1a). Next, we scanned the brains of the animals for induction of the immediate early gene *Fos* as a proxy for neural activity^30^ (see Methods for details). Our results showed stimulus-evoked labelling in the area postrema and strong labelling in the caudal nucleus of the solitary tract (cNST)^15^ in the brainstem (Fig. 1b and Extended Data Fig. 1); minor labelling was observed in response to control saline injections. The area postrema is known to be activated by body malaise^17^, and hence it would be expected to exhibit some labelling. The cNST, on the other hand, is the primary target of the vagus nerve^2,31^ and functions as the major conduit in the body–brain axis.

**Extended Data Fig. 2:**
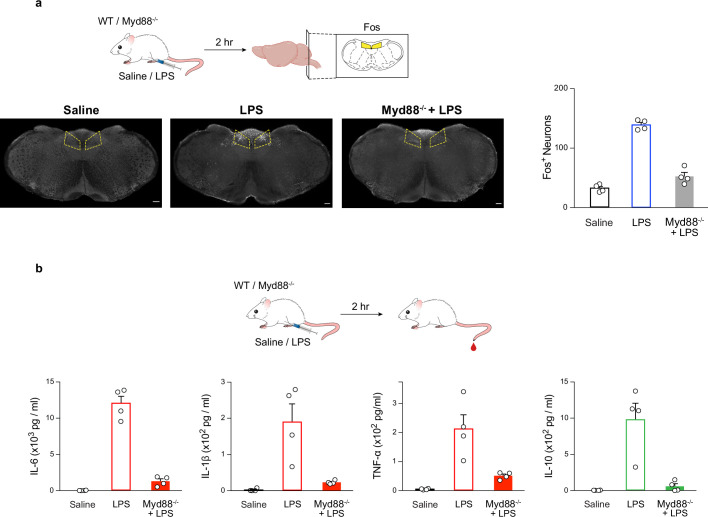
Normal Fos induction to LPS is lacking in the cNST of *Myd88* knockouts. **a**, Blocking LPS signaling abrogates Fos induction in response to LPS. WT and *Myd88*^−/−^ mice^57^ were injected with LPS intraperitoneally, and two hours later, brains were extracted, sliced and stained for Fos expression (see Fig. 1b). As a control, WT mice were injected with saline. Bilateral Fos expression is strongly induced by LPS in the cNST of WT mice but largely absent from *Myd88*^−/−^ mice; n = 4 mice each. The right panel shows the quantification of Fos-positive neurons. The equivalent area of the cNST (200 μm X 200 μm, bregma −7.5 mm) was processed, and positive neurons were counted. Values are means ± SEM; ANOVA with Tukey’s honestly significant difference (HSD) post hoc test, p < 0.0001 (Saline vs LPS); p < 0.0001 (LPS vs *Myd88*^−/−^ + LPS). Scale bar, 200 μm. **b**, *Myd88* knockouts have impaired cytokine responses to LPS^32^. WT and *Myd88*^−/−^ mice received an intraperitoneal injection of LPS, and peripheral blood was taken 2 h later to measure circulating levels of pro- inflammatory (IL-6, IL-1β, TNF-α) and anti-inflammatory (IL-10) cytokines by ELISA. As a control, WT mice were injected with saline. Note that cytokine induction is dramatically reduced in *Myd88*^−/−^ mice. n = 4 mice each group. Values are means ± SEM; ANOVA with Tukey’s HSD post hoc test, LPS vs *Myd88*^−/−^ + LPS: p < 0.0001 (IL-6); p < 0.01 (IL-1β); p < 0.01 (TNF-α); p < 0.01 (IL-10). No significant difference was observed between Saline and *Myd88*^−/−^ + LPS: p = 0.19 (IL-6), p = 0.88 (IL-1β), p = 0.52 (TNF-α); p = 0.96 (IL-10). Schematics were created using BioRender (https://biorender.com).

Importantly, injection of LPS in animals with a homozygous knockout for *Myd88* (an essential component of the LPS receptor in immune cells^32^) did not activate cNST neurons (Extended Data Fig. 2), showing that LPS stimulates cNST labelling via its action on immune cells. Robust cNST labelling was also observed in response to various other immune insults (Extended Data Fig. 1c,d).

To directly monitor the activation of cNST neurons following the peripheral LPS challenge, we targeted cNST neurons with an adeno-associated virus (AAV) with a GCaMP6s construct^33^ (see Fig. 1 caption), so as to drive expression of the activity reporter in cNST neurons, and recorded responses in awake behaving animals using fibre photometry (Fig. 1c). Our results demonstrated cNST activation that tracks the emergence and the development of the innate immune response (compare Fig. 1c with Fig. 1a).

If peripheral inflammation is sensed and transmitted by the vagus nerve to the cNST, then blocking the transfer of vagal signals should abolish LPS-evoked neural activity in the cNST. Indeed, bilateral subdiaphragmatic transection of the vagus nerve^5,34^ eliminated cNST responses to LPS (Fig. 1c). These results substantiate the vagal–cNST immune axis and demonstrate that the LPS-evoked activity is not the result of LPS directly accessing cNST neurons.

## cNST silencing transforms body immunity

We anticipated that if the LPS-activated neurons in the cNST function as an essential circuit modulating peripheral immune responses, then blocking their activation should significantly affect the inflammatory response.

**Fig. 2:**
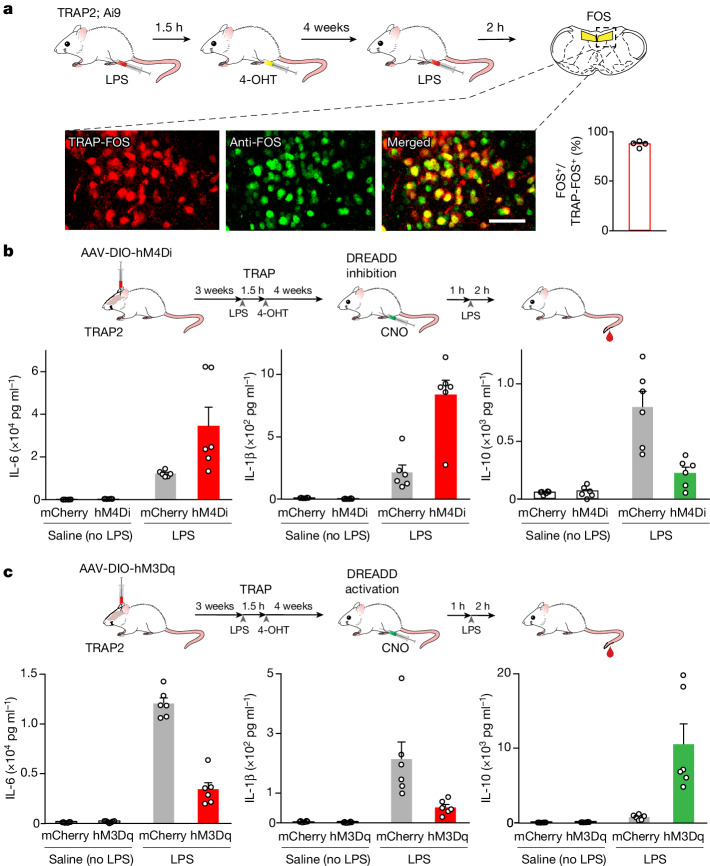
Removing brain regulation transforms the inflammatory response. **a**, Neurons marked by LPS-TRAPing (red, tdTomato) are the same as the FOS neurons labelled after a second cycle of LPS (green; see Methods). By comparing the number of neurons expressing tdTomato to the number of neurons labelled by FOS antibodies, we determined that more than 80% of LPS-TRAPed neurons were also positive for LPS–FOS (*n* = 4). Scale bar, 50 μm. 4-OHT, 4-hydroxytamoxifen. **b**, Inhibition of LPS-activated neurons in the cNST greatly increases the inflammatory response. AAVs carrying an mCherry construct, or the hM4Di inhibitory DREADD, were targeted to the cNST of LPS-TRAP2 mice for chemogenetic silencing. Control mCherry animals injected with LPS (grey bars) exhibit the expected induction of cytokines. By contrast, animals with silenced cNST neurons displayed increases in the levels of pro-inflammatory cytokines and a large reduction in the levels of an anti-inflammatory cytokine (IL-10; compare red or green and grey bars). Mice in all groups were given CNO 1 h before either the saline or the LPS injection; *n* = 6 for each group. Values are mean ± s.e.m.; Mann–Whitney *U*-tests: *P* = 0.24 (saline, IL-6), *P* = 0.97 (saline, IL-1β), *P* = 0.78 (saline, IL-10), *P* = 0.004 (LPS, IL-6), *P* = 0.004 (LPS, IL-1β) and *P* = 0.002 (LPS, IL-10). **c**, Chemogenetic activation of the cNST neurons during an immune response suppresses inflammation. The levels of anti-inflammatory (IL-10) and pro-inflammatory (IL-6 and IL-1β) cytokines in mice expressing excitatory DREADD (hM3Dq), or mCherry, in response to LPS are shown. All animals were given CNO 1 h before either the saline or the LPS injection (*n* = 6 for each group). Values are mean ± s.e.m.; Mann–Whitney *U*-tests: *P* = 0.17 (saline, IL-6), *P* = 0.93 (saline, IL-1β), *P* = 0.37 (saline, IL-10), *P* = 0.002 (LPS, IL-6), *P* = 0.002 (LPS, IL-1β) and *P* = 0.002 (LPS, IL-10). Schematics were created using BioRender (https://biorender.com).

We used the targeted recombination in active populations (TRAP) system^35^ to target Cre-recombinase to the LPS-activated neurons (Extended Data Fig. 1e,f) and a Cre-dependent genetic silencer to examine LPS-evoked responses in control and silenced animals. First, to monitor the fidelity of the TRAP strategy, we confirmed that the LPS-activated cNST neurons marked by the expression of FOS are the same as the neurons labelled by Cre-recombinase in the genetic TRAPing experiments. We genetically labelled the LPS-induced TRAPed neurons with a Cre-dependent tdTomato reporter, and then performed a second cycle of LPS stimulation followed by FOS antibody labelling. Our results confirmed that the majority (more than 80%) of the LPS-TRAPed neurons (that is, labelled with tdTomato in the cNST) were indeed co-labelled with the FOS antibodies in response to the second cycle of LPS stimulation (Fig. 2a).

Next, we bilaterally injected the cNST of LPS-TRAPed animals with an AAV carrying a Cre-dependent inhibitory DREADD^36^ (iDREADD; see Methods for details). Hence, the TRAPed LPS-activated neurons would turn-on Cre-recombinase and enable expression of the Cre-dependent iDREADD, thus allowing chemogenetic inhibition of those cells. The iDREADD-expressing animals were then challenged with LPS, and we monitored the resulting immune response (Fig. 2b, top panel). Chemogenetic inhibition of the cNST neurons resulted in a dramatic increase in the pro-inflammatory response and a concomitant decrease of the anti-inflammatory response (Fig. 2b, bottom panels); in essence, a run-away, out-of-control inflammatory response. Indeed, the levels of pro-inflammatory cytokines rise to over 300% compared with the levels observed in LPS-treated but not silenced animals (for example, IL-1β goes from 200 pg ml^−1^ to 800 pg ml^−1^; Fig. 2b), whereas the anti-inflammatory component exhibited a profound reduction (IL-10 levels were reduced from 750 pg ml^−1^ to approximately 250 pg ml^−1^; Fig. 2b). These results suggest that the cNST functions as a homeostatic neural control of peripheral immune responses.

## cNST activation suppresses inflammation

**Extended Data Fig. 3:**
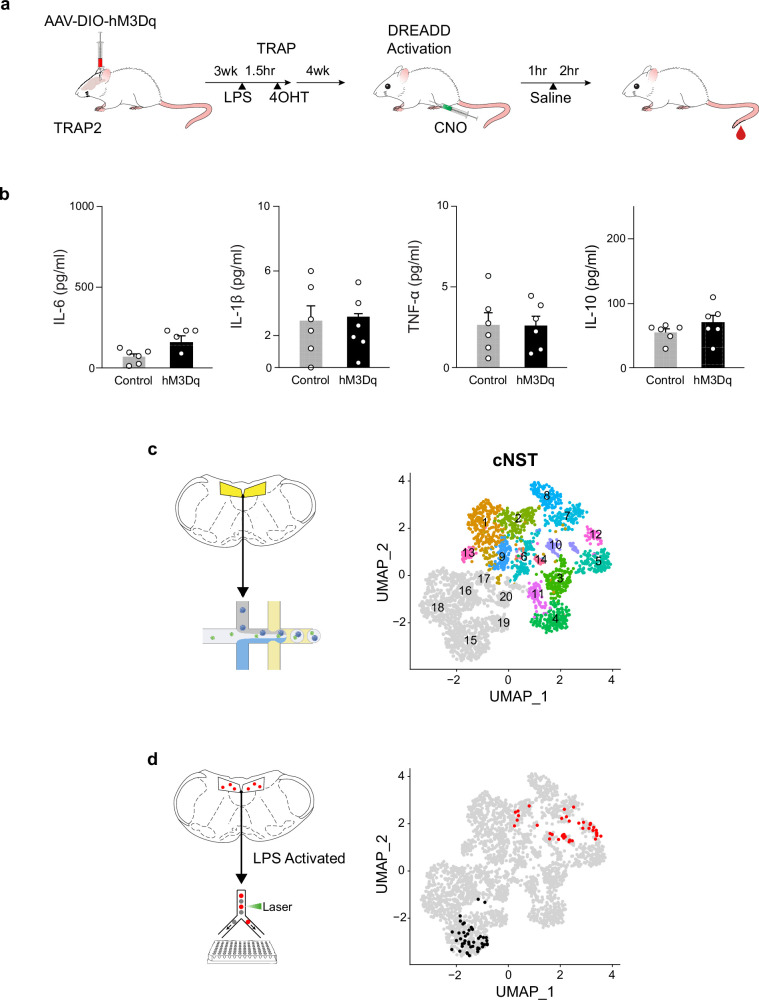
Activation of LPS-TRAPed neurons in the cNST does not elicit immune responses in the absence of immune challenge. **a**, Schematic of chemogenetic activation strategy. AAV viruses carrying a control mCherry construct, or the hM3Dq excitatory DREADD, were targeted to the cNST of TRAP2 mice for chemogenetic activation. Mice were TRAPed with LPS (50 μg kg^−1^). After 4 weeks, cytokine responses to saline (i.e., without LPS) was quantified in the presence of DREADD agonist, CNO. **b**, Shown are levels of anti-inflammatory (IL-10) and pro-inflammatory (IL-6, IL-1β, TNF-α) cytokines in the peripheral blood of mice expressing excitatory DREADD (hM3Dq), or control (mCherry), in the LPS-TRAPed cNST neurons. All mice were injected with CNO 1 h prior to saline stimulation. Part of the data presented comes from Fig. 2c and replotted with an expanded y axis. Note that in the absence of the immune stimuli (LPS), activation of this circuit produces no meaningful effect on circulating cytokine levels. Grey bars (control), TRAP2 animals injected with DIO-mCherry (n = 6); black bars (hM3Dq), TRAP2 animals injected with DIO-hM3Dq (n = 6). Values are means ± SEM; Mann–Whitney U-tests, p = 0.17 (IL-6), p = 0.93 (IL-1β), p = 0.93 (TNF-α), p = 0.37 (IL-10). **c**, Single-cell RNA sequencing (scRNA-seq) cataloging neuronal clusters in the cNST. A uniform manifold approximation and projection (UMAP) plot of transcriptomic data revealed 14 Glutamatergic neuronal clusters (1-14, colored) and 6 GABAergic clusters (15-20, grey). **d**, ScRNA-seq of individual LPS-TRAPed neurons from the cNST. The tdTomato-labeled LPS-TRAPed cells were isolated by FACS and individually sequenced. The UMAP of LPS-TRAPed neurons was then superimposed onto the UMAP of the entire cNST map, showing that in addition to excitatory clusters (7, 10, 12, red; see Fig. 3b), an inhibitory cluster (15, black) also contains LPS-TRAPed neurons. Activation of this inhibitory cluster has no effect on cytokine levels after LPS injection (see Extended Data Fig. 4 below). Schematics in panels **a**,**c**,**d** were created using BioRender (https://biorender.com).

Given that silencing LPS-activated neurons in the cNST leads to greatly intensified inflammation, we hypothesized that artificial activation of this circuit should produce the opposite effect, and thus suppress inflammation. We used the TRAP system to virally target an excitatory, rather than inhibitory, DREADD (hM3Dq)^36^ to the LPS-activated neurons, and tested the effect of activation of this circuit on the LPS-evoked inflammatory response. As predicted, chemogenetic activation of the LPS-TRAPed neurons inhibited the pro-inflammatory response while substantially increasing the anti-inflammatory response. As shown in Fig. 2c, the levels of pro-inflammatory cytokines were reduced by nearly 70% from the levels observed in the control LPS-evoked responses, whereas anti-inflammatory levels were up nearly tenfold. Together, these silencing and activation experiments demonstrate that modulating the activity of these brainstem neurons can bidirectionally regulate peripheral inflammation. Activating this circuit in the absence of an immune challenge has no effect on cytokine levels, validating its role in monitoring and regulating an immune response rather than initiating it (for example, no LPS control in Fig. 2c and Extended Data Fig. 3a,b).

## cNST neurons suppressing inflammation

**Fig. 3:**
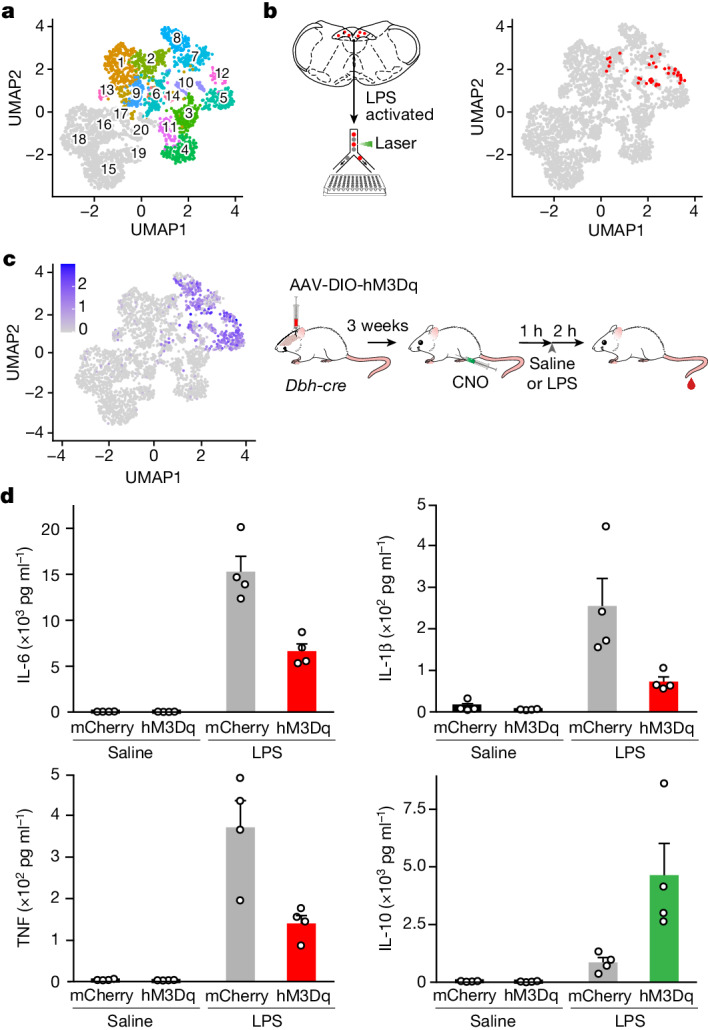
A genetically defined population of cNST neurons modulates body immunity. **a**, scRNA-seq cataloguing neuronal clusters in the cNST. A uniform manifold approximation and projection (UMAP) plot of transcriptomic data reveals 14 glutamatergic neuronal clusters (1–14, in colour) and 6 GABAergic clusters (15–20, in grey). **b**, scRNA-seq of individual LPS-TRAPed neurons from the cNST. The tdTomato-labelled LPS-TRAPed cells were isolated by fluorescence-activated cell sorting and individually sequenced. The UMAP of LPS-TRAPed neurons was then superimposed to the cNST map, showing the LPS-TRAPed neurons (highlighted in red). **c**, UMAP plot showing the normalized expression of the *Dbh* gene (left) and the strategy for hM3Dq DREADD activation of the DBH-expressing cNST neurons (right). **d**, Chemogenetic activation of DBH cNST neurons suppresses inflammation. The levels of anti-inflammatory (IL-10) and pro-inflammatory (IL-6, IL-1β and TNF) cytokines in mice expressing either excitatory hM3Dq or mCherry 2 h after LPS stimulation are shown. All mice were given CNO 1 h before the injection of saline or LPS. *n* = 4 animals for each group. Note the major decrease in the levels of pro-inflammatory cytokines and the large increase in the levels of anti-inflammatory IL-10. Values are mean ± s.e.m.; Mann–Whitney *U*-tests: *P* = 0.08 (saline, IL-6), *P* = 0.20 (saline, IL-1β), *P* = 0.23 (saline, TNF), *P* = 0.77 (saline, IL-10), *P* = 0.03 (LPS, IL-6), *P* = 0.03 (LPS, IL-1β), *P* = 0.03 (LPS, TNF) and *P* = 0.03 (LPS, IL-10). Schematics in panels **b**,**c** were created using BioRender (https://biorender.com).

To identify the cNST neurons modulating inflammation, we performed single-cell RNA sequencing (scRNA-seq) on 4,008 cells from the cNST (Fig. 3a). We then carried out scRNA-seq on 288 individual neurons TRAPed with LPS (along with approximately 100 unlabelled neurons) and showed that the LPS-TRAPed neurons are primarily found in three related glutamatergic clusters (clusters 7, 10 and 12, with a small number in cluster 2) (Fig. 3b) and one GABAergic cluster (cluster 15) (Extended Data Fig. 3c,d).

**Extended Data Fig. 4:**
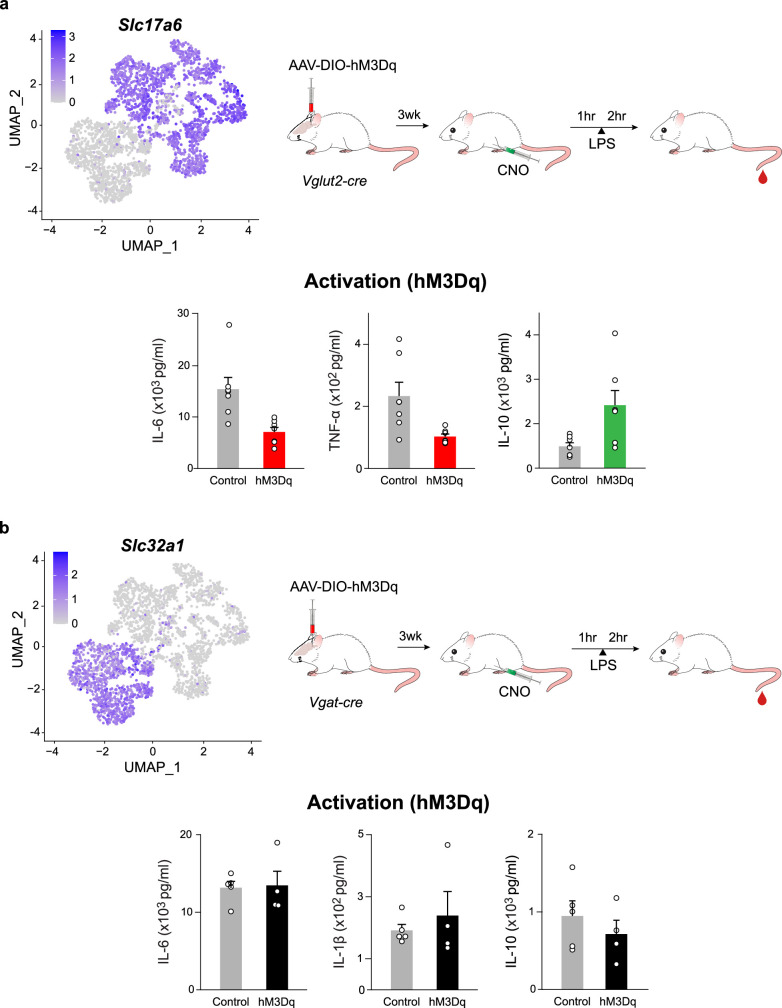
Activation of excitatory but not inhibitory neurons suppresses LPS-induced inflammation. **a**, Activation of cNST glutamatergic neurons suppresses LPS-induced inflammation. Upper panels, UMAP plot of the normalized expression of *Slc17a6* (also known as *Vglut2*) highlighting glutamatergic (excitatory) neuronal clusters in the cNST; also illustrated is the strategy for chemogenetic activation of the excitatory cNST neurons. An AAV virus carrying the Cre-dependent excitatory DREADD (hM3Dq) was targeted bilaterally to the cNST of *Vglut2-cre* mice. lower panels, shown are circulating levels of anti-inflammatory (IL-10) and pro-inflammatory (IL-6, TNF-α) cytokines in the peripheral blood of LPS-stimulated mice expressing excitatory DREADD (hM3Dq), or control (mCherry), in glutamatergic neurons. All animals were given CNO 1 h prior to the LPS injection. n = 7 animals for each group. Values are means ± SEM; Mann–Whitney U-tests, p = 0.002 (IL-6), p = 0.004 (TNF-α), p = 0.02 (IL-10). Note the increase in the levels of anti-inflammatory (compare grey and green bars), and decrease in the levels of pro-inflammatory cytokines (compare grey and red bars). **b**, Upper panels, UMAP plot of the normalized expression of *Slc32a1* (also known as *Vgat*) highlighting the GABAergic (inhibitory) neuronal clusters in the cNST, and the chemogenetic strategy for activation of the inhibitory cNST neurons. Lower panels, shown are levels of anti-inflammatory (IL-10) and pro-inflammatory (IL-6, IL-1β) cytokines in the peripheral blood of mice after LPS-stimulation, both in mice expressing excitatory DREADD (hM3Dq), or control (mCherry) in GABAergic neurons. n = 4 mice for hM3Dq group and 5 mice for control (mCherry) group. Values are means ± SEM; Mann–Whitney U-tests, p = 0.73 (IL-6), p = 0.90 (IL-1β), p = 0.73 (IL-10). Note that activation of cNST GABAergic neurons does not meaningfully impact LPS-induced inflammation. Schematics were created using BioRender (https://biorender.com).

**Extended Data Fig. 5:**
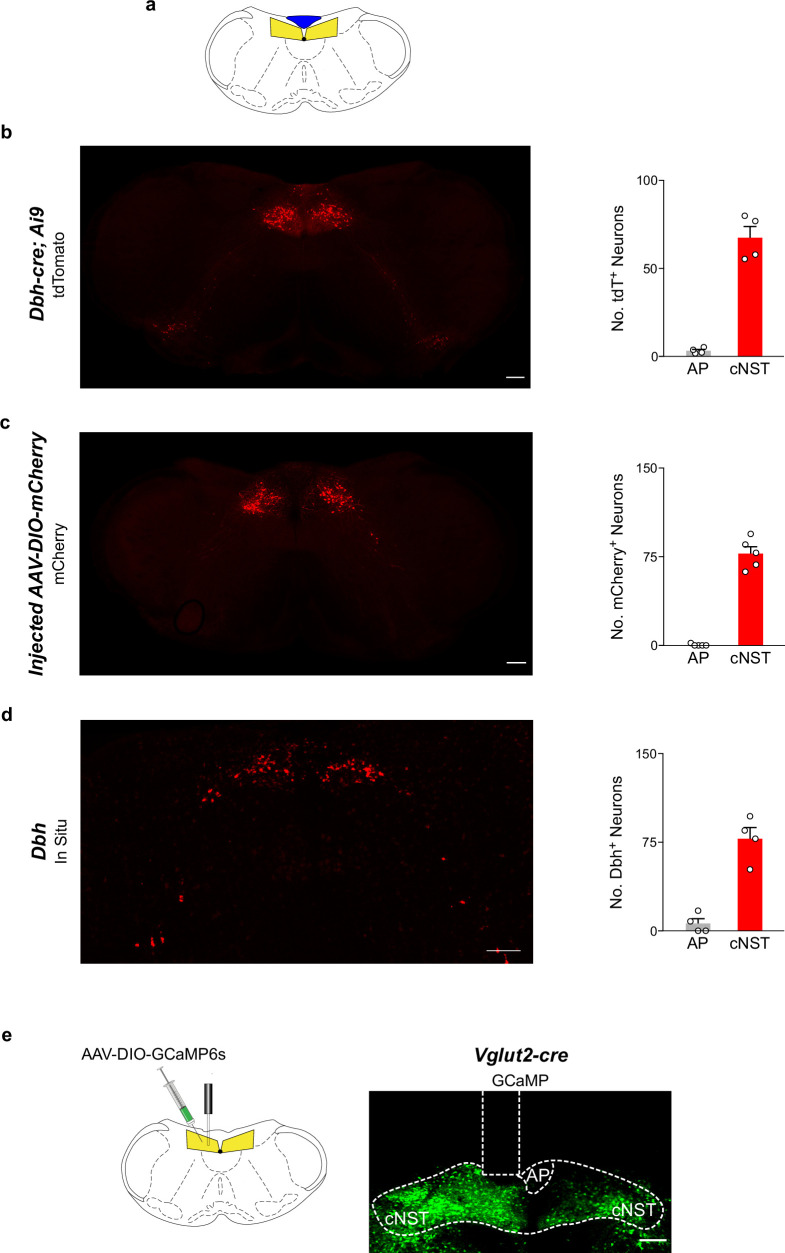
DBH is selectively expressed in the cNST. A previous study^15^ reported that DBH was expressed almost exclusively in the AP. However, this conclusion was based solely on tissue extraction and sequencing, without anatomical validation. By contrast, we validated expression by directly examining DBH-expressing neurons in the cNST and in the AP. **a**, Diagram of a coronal section highlighting the cNST (in yellow) and the AP (in blue). **b-d**, First, we examined DBH expression by using *Dbh-cre* mice, crossed to the Ai9 tdT-reporter line^63^. We detected most labeling in the cNST (n = 4 mice), with very minimal expression in the AP in the adult brain, and some of this may reflect limited expression during development (i.e., the Cre reporter acting as a lineage tracer). Next, we directly injected a Cre-dependent mCherry-reporter virus (AAV9-Syn-DIO-mCherry) into the cNST and AP of adult mice, and indeed nearly all of the labeling is detected in the cNST, with almost no expression in the AP (n = 5 mice). Finally, we performed in-situ hybridizations, and as shown with the reporter mice, expression is largely restricted to the cNST, with very low levels in the AP. Scale bars, 200 μm. Shown in the bar graphs are the quantitation of DBH expressing neurons in cNST vs AP. **e**, Sample brain demonstrating expression of GCaMP6s restricted to the cNST, with minimal expression in the AP; the image also demarks the location of the recording fiber (dashed rectangle). Scale bar, 100 μm. Similar results were observed in the analyzed animals, both control and vagotomized (n = 6 each). Schematics in panels **a**,**e** were created using BioRender (https://biorender.com).

**Extended Data Fig. 6:**
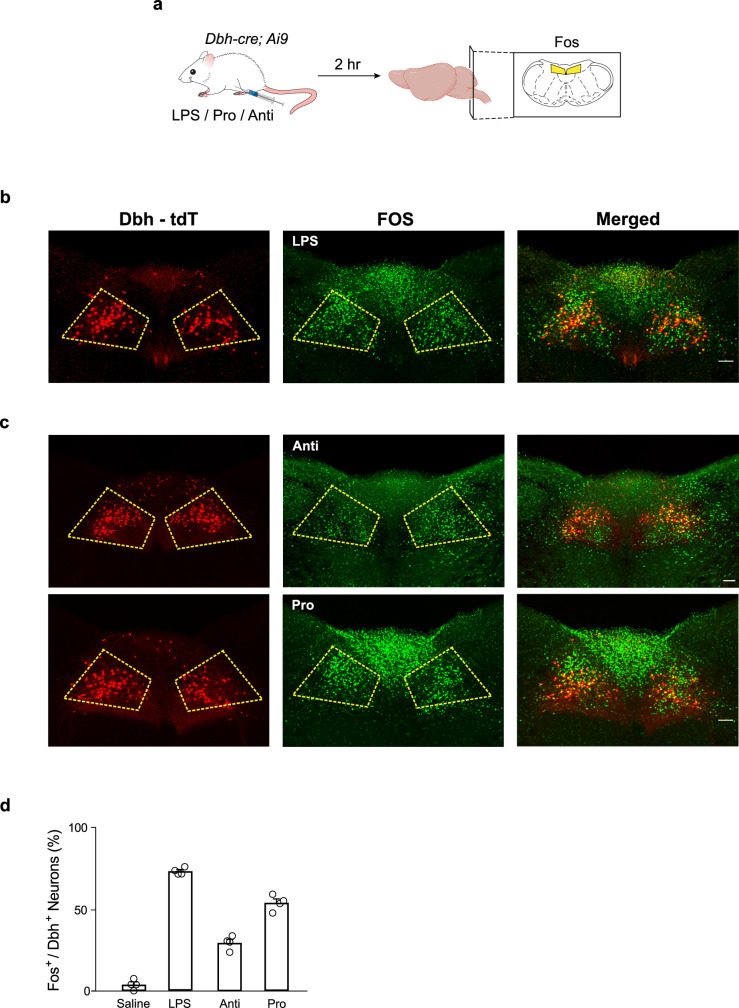
Immune insult activate DBH neurons in the cNST. **a**, Schematic illustrating Fos induction in DBH neurons in the cNST by LPS and cytokine stimulation. *Dbh-cre* mice were injected intraperitoneally with LPS, IL-10 or a cocktail of IL-6, IL-1β, and TNF-α, and brain slices were analyzed for Fos and *Dbh* labeling. DBH neurons were marked by tdTomato (tdT) expression (Ai9 reporter line^63^), and Fos by immunohistochemistry. Schematics in panel **a** were created using BioRender (https://biorender.com). **b-c**, Coronal sections of the brain stem showing neurons expressing DBH (Dbh-tdT, red) and neurons activated by LPS (top row), IL-10 (anti-inflammatory, middle row), or by a cocktail of 3 pro-inflammatory cytokines (100 μg kg^−1^ TNF-α,100 μg kg^−1^ IL-6, 100 μg kg^−1^, IL-1β, bottom row). Note that all three stimuli activate DBH neurons. Scale bar, 200 μm. **d**, Quantification of the fraction of DBH neurons that express immune-induced Fos (n = 4 mice each group). Anti = IL-10, pro = a mixture of TNF-α, IL-6, and IL-1β. Values are means ± SEM; ANOVA with Tukey’s HSD post hoc test, LPS vs Saline: p < 0.0001; Anti vs Saline: p < 0.0001; Pro vs Saline: p < 0.0001. The fraction of Fos+ neurons that also expressed DBH are: LPS 21.4% ± 1.5%; Pro 20.0% ± 1.2%; Anti 33.3% ± 3.6%. As would be expected, there are significantly more Fos-positive neurons activated by the immune stimuli than the overlap with DBH; these likely respond and/or mediate other effects of LPS and cytokine stimulation (like malaise, etc).

**Extended Data Fig. 7:**
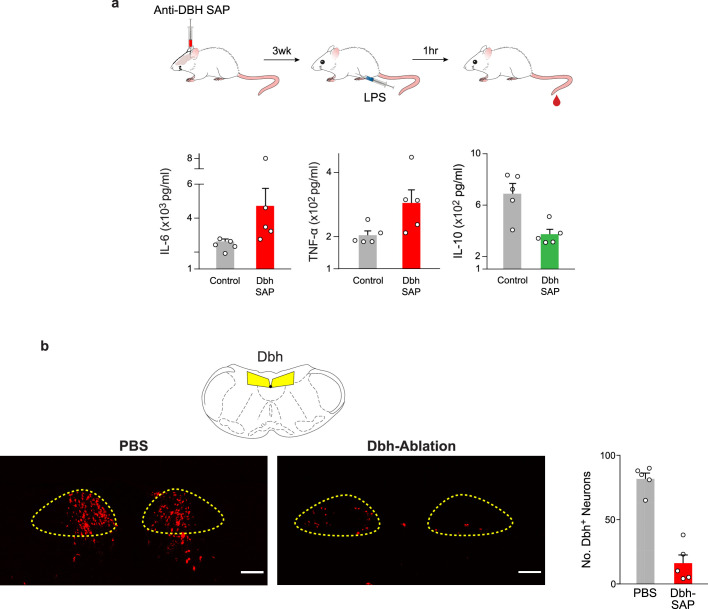
Ablation of DBH cNST neurons increases inflammatory responses. **a**, Anti-DBH Saporin (SAP)^72,73^ was injected bilaterally into the cNST to selectively ablate DBH neurons; control mice were injected with PBS. The bar graphs show circulating levels of pro-inflammatory (IL-6, TNF-α) and anti-inflammatory (IL-10) cytokines in the peripheral blood of control mice, and DBH-ablated animals (Dbh-SAP) after LPS stimulation. Note the significant increase in the levels of IL-6 and TNF-α after LPS stimulation in Anti-DBH Saporin mice versus control animals (n = 5 each group). As seen with the TRAPped cNST neurons (Fig. 2b), the level of IL-10 is greatly reduced in the ablated mice (n = 5 each group). Values are means ± SEM; Mann–Whitney U-tests, IL-6: p = 0.02; TNF-α: p = 0.04; IL-10: p = 0.02. **b**, Loss of DBH neurons in the cNST after Dbh-SAP induced cell-death. Upper panel, diagram of a coronal section highlighting the cNST (in yellow). Lower panels show in situ hybridization signals for *Dbh* RNA in the cNST of control and Dbh-SAP treated mice. Note the dramatic loss of DBH neurons in the cNST of the experimental animals (compare right panel with left control); similar results were observed in independently injected animals; the bar graph shows quantitation for 5 animals. PBS, control animals injected with PBS; Dbh-Ablation, animals injected with Dbh-SAP (see Methods for details). Scale bars: 200 μm. Values are means ± SEM; Mann–Whitney U-tests, p = 0.008. Schematics were created using BioRender (https://biorender.com).

We next tested whether chemogenetic activation of the excitatory (glutamatergic) or inhibitory (GABAergic) neurons could alter LPS-induced responses. We injected AAVs carrying a Cre-dependent excitatory DREADD into the cNST of either *Vglut2-cre* or *Vgat-cre* mice. Our results showed that activation of excitatory, but not inhibitory, neurons effectively suppressed LPS-induced inflammation and largely mirrored the results obtained following activation of the LPS-TRAPed neurons (Extended Data Fig. 4a); no effect was observed when activating GABAergic neurons (Extended Data Fig. 4b). Next, we screened clusters 7, 10 and 12 for common, selectively expressed genes and identified the dopamine β-hydroxylase (*Dbh*)^37^ gene as a candidate marker (Fig. 3c). In contrast to previous reports^15^, DBH-expressing neurons in the brainstem are almost exclusively located in the cNST (see Extended Data Fig. 5 for details) and are strongly activated in response to LPS (Extended Data Fig. 6). We obtained *Dbh-cre* mice^38^ and targeted their cNST with an AAV encoding a Cre-dependent excitatory DREADD^36^. As anticipated, activation of DBH-expressing neurons in the cNST markedly suppressed pro-inflammatory cytokines while greatly enhancing the anti-inflammatory IL-10 levels (Fig. 3c,d), demonstrating the ability of these cNST neurons to drive immune suppression. Next, we ablated the DBH^+^ neurons in the cNST and, as expected (Fig. 2), we observed dysregulation of the immune response (Extended Data Fig. 7).

## Vagal responses to immune cytokines

How do cNST neurons monitor peripheral immune activity to instruct appropriate immune modulation? Given that information is being transferred via the vagal body–brain axis (Fig. 1c), we reasoned that specific vagal neurons may respond to cytokines released during LPS-induced inflammation and inform the brain of the emerging immune response.

**Fig. 4:**
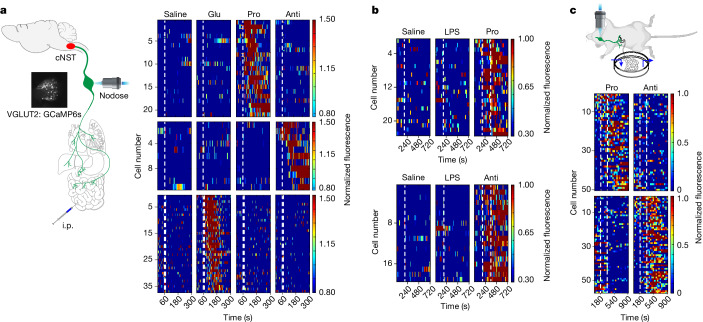
Vagal neurons responding to anti-inflammatory and pro-inflammatory cytokines. **a**, Recording of calcium responses in vagal neurons expressing GCaMP6s while stimulating mice with cytokines intraperitoneally. The heatmaps depict *z*-score-normalized fluorescence traces from two non-overlapping populations of neurons: responders to pro-inflammatory (Pro) cytokines (top panels) and responders to anti-inflammatory (Anti) cytokines (middle panels). Each row represents the activity of a single cell over 5 min. Stimulus was given at 60 s (dashed line). *n* = 5 mice, TNF (3 mice), IL-1β (2 mice) and IL-10 (5 mice); 21 of 423 imaged neurons responded to pro-inflammatory stimuli (13 to TNF and 8 to IL-1β), and 11 of 423 responded to IL-10. As positive controls, we used intestinal stimulation with glucose (Glu; 10 s); this activates the gut–brain axis^5,6^, but stimulates different vagal neurons (lower panels). These imaging experiments used cytokine concentrations that were lower or comparable with that measured during LPS-induced inflammation (see Extended Data Fig. 10b). The overall percent of responding neurons is similar to what is observed for vagal neurons dedicated to other body–brain signalling pathways^2,6^. i.p., intraperitoneal. **b**, Vagal neurons are not directly activated by LPS, even when using high concentrations of LPS (0.5 mg kg^−1^; *n* = 5 mice; pro: TNF; anti: IL-10). **c**, We carried out a similar experiment by using a perfusion chamber rather than intraperitoneal injections of LPS (see Methods). Each row in the heatmaps represents the averaged activity of a single cell to two trials. The dashed lines denote stimulus time window (180 s). *n* = 7 for IL-1β, *n* = 12 for IL-6 and *n* = 19 for IL-10. See also Extended Data Fig. 8. Schematics in panels **a**,**c** were created using BioRender (https://biorender.com).

We implemented an in vivo calcium imaging platform^6^ to record immune-evoked neural activity in the nodose (vagal) ganglia where the cell bodies of vagal sensory neurons reside, while animals were challenged with different cytokines. We targeted the calcium indicator GCaMP6s^33^ to all vagal sensory neurons using a *Vglut2-cre* driver, and used a one-photon functional imaging setup to record real-time vagal neuron responses^6^ to cytokine stimuli delivered intraperitoneally. As control, we also imaged responses to LPS and to intestinal delivery of sugar, a stimulus known to activate the nutrient-sensing, gut–brain axis, via a specific population of vagal neurons^5,6^. Our results showed that anti-inflammatory and pro-inflammatory cytokines activate two discrete non-overlapping populations of vagal sensory neurons, each accounting for a small fraction of all nodose ganglion neurons (Fig. 4a; see the legend). As anticipated, these do not overlap with the sugar-sensing vagal neurons^8^ (Fig. 4a, bottom panel). Importantly, LPS does not directly activate vagal neurons (Fig. 4b).

**Extended Data Fig. 8:**
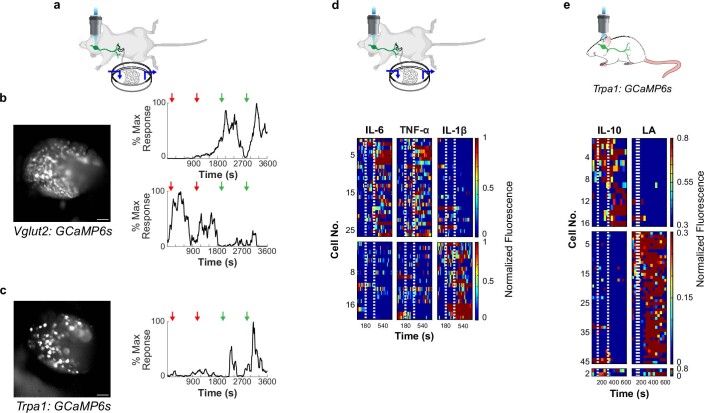
Vagal responses to anti-inflammatory and pro-inflammatory cytokines. **a**, Schematic of vagal calcium imaging while simultaneously delivering cytokines onto the intestines. (see Methods for details) **b**, The micrograph shows a representative view of a nodose ganglion from *Vglut2-cre*; *Ai96* during an imaging session. All vagal sensory neurons express GCaMP6s. Right panels show representative traces from vagal neurons selectively responding to anti-inflammatory (IL-10, upper panel) and pro-inflammatory (IL-6, lower panel) cytokines. Each cytokine was perfused for 180 seconds (starting at the time indicated by the color arrows; green, IL-10; red, IL-6) in 2 repeat trials. Scale bar, 100 μm. Summary data is presented in Fig. 4c. **c**, Responses of TRPA1 vagal neurons to anti-inflammatory cytokines. The micrograph depicts a sample nodose ganglion from *Trpa1-cre*; *Ai162* during an imaging session. Right panel is the sample trace. Scale bar, 100 μm. **d**, The heat maps depict z-score-normalized fluorescence traces from vagal neurons responding to individual pro-inflammatory cytokines. Each row represents the averaged activity of a single cell to 2 trials. Dashed lines denote stimulus time window (180 sec). n = 5 mice. **e**, IL-10 and fat activate distinct subsets of TRPA1 vagal neurons. Previously, we showed that a subset of TRPA1-vagal neurons transfer fat signals from the intestines to the brain, via the gut-brain axis, to drive the development of fat preference^5^. The heat map shows that the TRPA1 neurons that selectively responded to extraintestinal application of IL-10 (top panel), are unique and separate from the pool of neurons that responded to fat (LA, middle panel). Shown are the responses of 63 TRPA1-labeled vagal neurons to anti-inflammatory stimuli and to intestinal delivery of fat^5^. Heat maps depict z-score-normalized responses to stimuli of IL-10 (1 *μ*g ml^−1^) and fat (LA, 10% linoleic acid). IL-10 was perfused onto the intestines for 180 s (dashed lines) and linoleic acid was infused into the gut for 10 s (dashed lines)^5^. Each row represents the average activity of a different neuron during two exposures to the stimulus. n = 4 mice. Shown also are 2 neurons that appeared to respond to both stimuli (bottom panel); given that these represent less than 1 neuron per animal they were not considered further. Schematics in panels **a**,**d**,**e** were created using BioRender (https://biorender.com).

Because the delivery of cytokines via intraperitoneal injections limits the ability to examine responses across repeat trials in the same animal, we implemented an in vivo preparation that enables repeated perfusion of cytokines over time (see Methods for details). As the small intestines are a major substrate of vagal innervation^39^ and house a vast reservoir of immune cells^40^ capable of releasing cytokines in response to LPS stimulation, we anticipated that this would provide an effective strategy. As expected, our results demonstrated reproducible vagal responses to cytokine stimulation (Fig. 4c and Extended Data Fig. 8), thus substantiating the proposal that cytokines themselves function as an immune mediator in the body–brain axis, with the vagal neurons functioning as the conduit transmitting the inflammatory information to the cNST. Two direct predictions emerge from these results. First, injection of cytokines should activate the cNST DBH neurons (Extended Data Fig. 6c,d), and second, activating the selective vagal neurons should modulate the immune response, much like activating the cNST target neurons (see below).

## Vagal activation by inflammatory signals

**Fig. 5:**
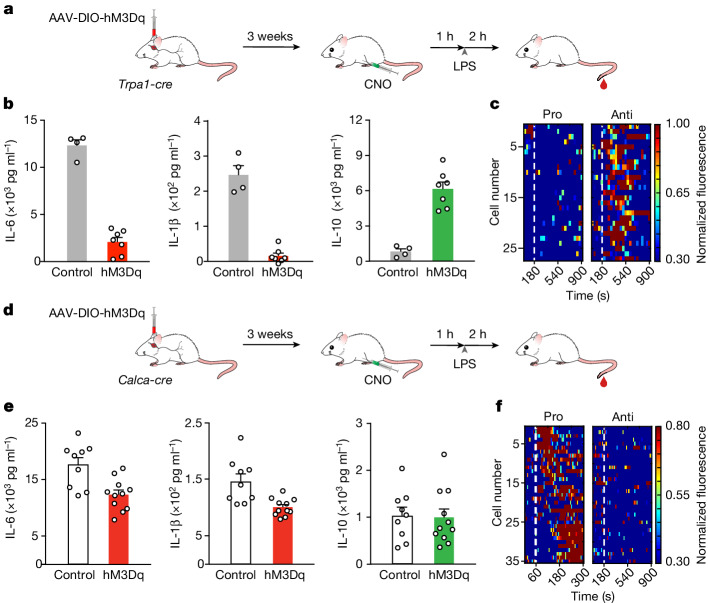
Vagal control of inflammation. **a**, Chemogenetic activation of TRPA1 vagal neurons. hM3Dq was targeted bilaterally to the nodose ganglion of *Trpa1-cre* mice^5^. Control animals received AAV-DIO-mCherry. **b**, Chemogenetic activation of TRPA1 vagal neurons suppresses inflammation. The levels of IL-6, IL-1β and IL-10 cytokines in mice expressing hM3Dq (*n* = 7 mice) and mCherry (*n* = 4 mice) are shown. Blood was collected 2 h after LPS, and all animals were given CNO 1 h before LPS injection. Values are mean ± s.e.m.; Mann–Whitney *U*-tests: *P* < 0.01 (IL-6), *P* < 0.01 (IL-1β) and *P* < 0.01 (IL-10). **c**, Heatmaps depict *z*-score-normalized fluorescence traces from IL-10-responding TRPA1 vagal neurons. Each row represents the activity of a single cell over 15 min. The experiment was carried out using intraperitoneal injection or perfusion with similar results. *n* = 6 mice. Pro: IL-6, anti: IL-10. A total of 27 of 189 imaged TRPA1 neurons responded to IL-10. **d**, Chemogenetic activation of CALCA vagal neurons. AAV-DIO-hM3Dq was targeted bilaterally to the nodose ganglion of *Calca-cre* mice^45^. Controls received AAV-DIO-mCherry. **e**, Chemogenetic activation of CALCA vagal neurons reduces the levels of pro-inflammatory cytokines. The levels of anti-inflammatory (IL-10) and pro-inflammatory cytokines (IL-6 and IL-1β) in mice expressing hM3Dq (*n* = 11 mice) and mCherry (*n* = 9 mice) are shown. Blood samples were collected 2 h after LPS stimulation, and all animals were given CNO 1 h before injection of LPS. Values are mean ± s.e.m.; Mann–Whitney *U*-tests: *P* < 0.01 (IL-6), *P* = 0.001 (IL-1β) and *P* = 0.88 (IL-10). **f**, Heatmaps depict *z*-score-normalized fluorescence traces from CALCA vagal neurons in response to pro-inflammatory cytokines (IL-6 and IL-1β). The experiment was carried out using intraperitoneal injections; *n* = 6 mice. A total of 35 of 211 imaged CALCA neurons responded to the pro-inflammatory stimuli. Schematics in panels **a**,**d** were created using BioRender (https://biorender.com).

**Extended Data Fig. 9:**
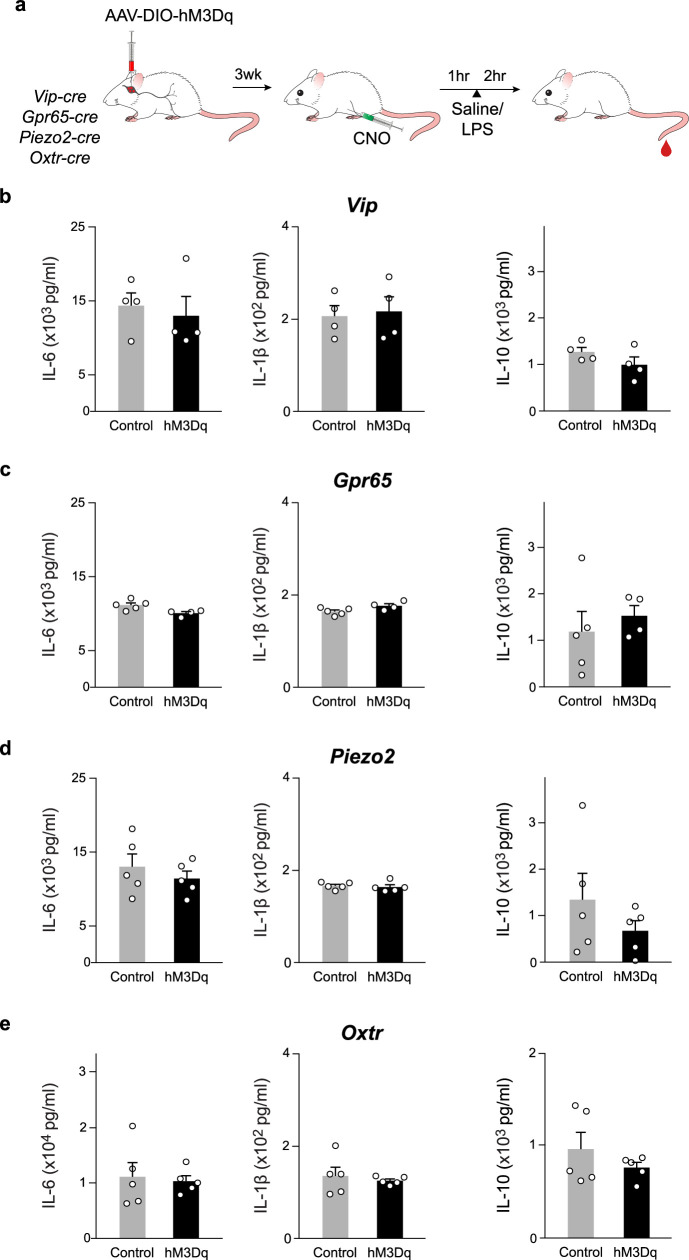
Neuronal clusters in the Vagal ganglia. **a**, Strategy for chemogenetic activation of vagal neuronal populations. An excitatory DREADD receptor (via AAV-DIO-hM3Dq) was targeted bilaterally to the nodose ganglia of *Vip-cre*, *Gpr65-cre*, *Piezo2-cre* and *Oxtr-cre* mice. The mice were then examined for changes in circulating cytokine levels in response to LPS in the presence of the DREADD receptor agonist CNO. Schematics in panel **a** were created using BioRender (https://biorender.com). **b-e**, The bar graphs show cytokine levels of IL-6, IL-1β and IL-10 in the peripheral blood of mice expressing either excitatory DREADD (hM3Dq) or control mCherry in VIP, GPR65, PIEZO2, OXTR vagal neurons, 2 h after LPS stimulation. All mice were injected with CNO 1 h prior to LPS. **b**, *Vip*: n = 4 each group; Mann–Whitney U-tests, p (IL-6) = 0.88, p (IL-1β) = 0.88, p (IL-10) = 0.2. **c**, *Gpr65*: n = 5 (control) and 4 (hM3Dq); Mann–Whitney U-tests, p (IL-6) = 0.03, p (IL-1β) = 0.06, p (IL-10) = 0.55. **d**, *Piezo2*: n = 5 each group; Mann–Whitney U-tests, p (IL-6) = 0.54, p (IL-1β) = 0.42, p (IL-10) = 0.42. **e**, *Oxtr*: n = 5 each group; Mann–Whitney U-tests, p (IL-6) = 0.84, p (IL-1β) = 0.65, p (IL-10) = 0.84. Values are means ± SEM; Activation of any of these vagal populations has no appreciable effect on LPS-induced cytokine responses.

**Extended Data Fig. 10:**
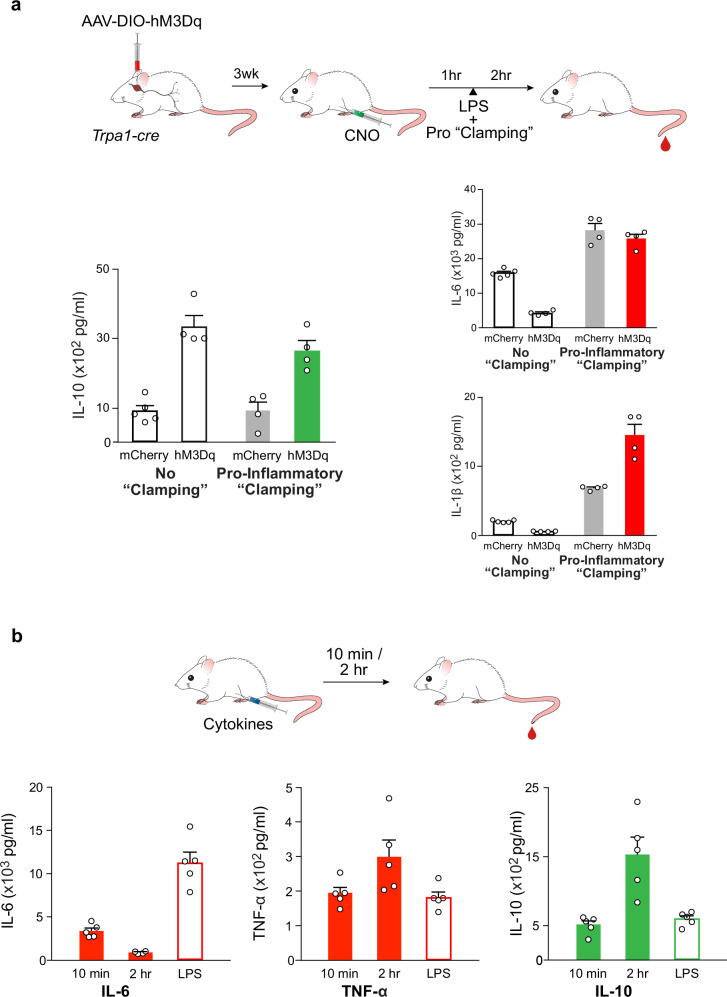
Enhancement of the anti-inflammatory response does not rely on the reduction of pro-inflammatory cytokines. **a**, AAV viruses carrying a control mCherry construct, or the hM3Dq excitatory DREADD, were targeted bilaterally to the nodose ganglion of *Trpa1-cre* mice for chemogenetic activation. All of the mice received an intraperitoneal injection of LPS to elicit an inflammatory response. Animals were then divided into 2 groups: control (no clamping), and the experimental (Pro-inflammatory clamping) where they were additionally injected with high levels of a pro-inflammatory cytokine cocktail (IL-6, IL-1β, TNF-α) to “clamp”, and thus maintain a high pro-inflammatory state (see Methods). Animals with activated TRPA1 vagal neurons in the control group (i.e., only injected with LPS) exhibited the expected enhancement in circulating IL-10 and a reduction in the levels of the pro-inflammatory cytokines. Notably, the levels of IL-10 remain similarly enhanced by TRPA1 vagal stimulation, even when the levels of pro-inflammatory cytokines are not suppressed. Blood samples were collected 2 h after LPS stimulation, and all animals were given CNO 1 hr prior to LPS injection. n = 5 mice (mCherry No “Clamping”); n = 4 for all other groups. Values are means ± SEM; Mann–Whitney U-tests, IL-10 levels in mCherry No “Clamping” vs mCherry Pro-inflammatory “Clamping”, p = 0.99; IL-10 levels in hM3Dq No “Clamping” vs hM3Dq Pro-inflammatory “Clamping”, p = 0.20. **b**, Levels of circulating IL-6, TNF-α, IL-10 in mice following an intraperitoneal injection of exogenous IL-6 (100 μg kg^−1^), TNF-α (100 μg kg^−1^), or IL-10 (100 μg kg^−1^), taken 10 mins or 2 hrs after the injection (n = 5 mice each group). Also shown are the levels of the same cytokines after LPS (2 hrs). Values are means ± SEM. Schematics were created using BioRender (https://biorender.com).

Because of the significance of suppressing an inflammatory state by modulating brain–body signals, we focused first on identifying vagal neurons mediating anti-inflammatory responses. Our strategy was to use the scRNA-seq cell atlas of the nodose ganglion^41–43^ to target excitatory DREADDs to different populations, and assess the effect of activation on LPS-induced immune responses. To ensure that only vagal neurons are activated in these experiments, we directly injected the AAV-DIO-hM3Dq (DREADD) virus bilaterally into the nodose ganglia of the various Cre-reporter mouse lines (Fig. 5 and Extended Data Fig. 9). Our results showed that activating the transient receptor potential ankyrin 1 (TRPA1)-expressing vagal neurons^5^ dramatically enhanced the anti-inflammatory response, and severely suppressed the levels of pro-inflammatory cytokines (Fig. 5a,b). Indeed, we observed a more than 80% decrease in the circulating levels of pro-inflammatory cytokines and a nearly sixfold increase in the levels of IL-10. We next explored whether this enhancement of the anti-inflammatory response depends on the reduction of the pro-inflammatory cytokines. We performed a ‘clamping-like’ experiment that artificially maintains pro-inflammatory cytokines at high levels and examined the anti-inflammatory response when activating the TRPA1 neurons. Our results demonstrated that, despite persistently high levels of pro-inflammatory cytokines, IL-10 was still dramatically enhanced in response to TRPA1 neuronal activation (Extended Data Fig. 10a).

**Extended Data Fig. 11:**
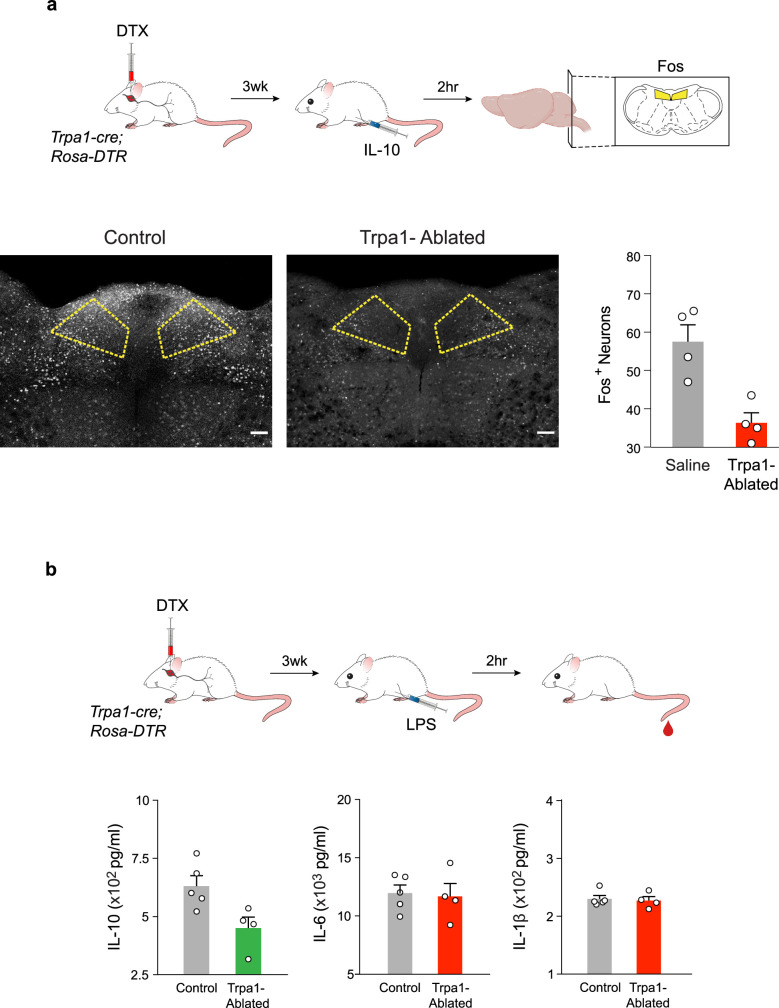
Ablation of TRPA1 vagal neurons prevents the emergence of a normal anti-inflammatory response. **a**, Ablation of TRPA1 vagal neurons block the induction of Fos in response to IL-10 stimulation. Diphtheria toxin (DTX) was injected bilaterally into the nodose ganglion of *Trpa1-cre;Rosa-DTR* mice^66^ to selectively ablate TRPA1 vagal neurons. Control animals received injection of PBS. Mice were then examined for cNST Fos induction 2 h following intraperitoneal injection of IL-10 (see Extended Data Fig. 6). IL-10 stimulation induces significant Fos labelling in control but not in animals lacking vagal TRPA1 neurons (Trpa1-Ablated). The right panel shows the quantification of Fos-positive neurons (n = 4 mice each). The equivalent area of the cNST (200 μm X 200 μm, bregma −7.5 mm) was processed. Values are means ± SEM; Mann–Whitney U-test, p = 0.03. **b**, Ablation of TRPA1 vagal neurons prevents a normal anti-inflammatory response. Bar graphs show the levels of anti-inflammatory (IL-10) and pro-inflammatory (IL-6, IL-1β) cytokines in the peripheral blood of control mice, and TRPA1-ablated animals (Trpa1-ablated) after LPS stimulation. Note the significant change in the levels of IL-10 after LPS stimulation in mice missing TRPA1 vagal neurons (n = 4) versus control animals (n = 5). By contrast, the levels of IL-6 and IL-1β are largely unaffected in mice lacking TRPA1 vagal neurons. Values are means ± SEM; Mann–Whitney U-tests, IL-10: p = 0.03; IL-6: p = 0.90; IL-1β: p = 0.90. Schematics were created using BioRender (https://biorender.com).

To define the response properties of the TRPA1-expressing vagal neurons, we targeted the expression of GCaMP6s^33^ and imaged their responses when the animals were challenged with anti-inflammatory or pro-inflammatory cytokines. Our experiments showed that IL-10, but not pro-inflammatory cytokines, activated the TRPA1-expressing vagal neurons (Fig. 5c and see also Extended Data Fig. 8e). Given these results, we hypothesized that removing the TRPA1-expressing vagal neurons from this circuit should prevent the transfer of anti-inflammatory signals to the brain. We genetically ablated TRPA1-expressing vagal neurons by targeting the diphteria toxin receptor^44^, and then challenged the animals with IL-10 or LPS. Indeed, our results demonstrated that the cNST was very poorly activated in response to injection of IL-10 (Extended Data Fig. 11a) and, more importantly, the anti-inflammatory response was severely truncated; IL-10 levels are only about 50% of what is observed in control animals after LPS stimulation, with no effect on the pro-inflammatory response (Extended Data Fig. 11b). These results reveal the TRPA1-expressing vagal neuron as a conduit for relaying anti-inflammatory signals via the body–brain axis to reinforce the anti-inflammatory state.

Next, we explored the vagal neurons responding to pro-inflammatory signals. Our experiments showed that calcitonin-related polypeptide-α (CALCA)-expressing neurons^45^ in the vagal ganglia responded selectively to pro-inflammatory stimuli (Fig. 5f) and their chemogenetic activation significantly altered the levels of circulating pro-inflammatory cytokines (Fig. 5d,e).

## A vagal–cNST body–brain circuit

**Extended Data Fig. 12:**
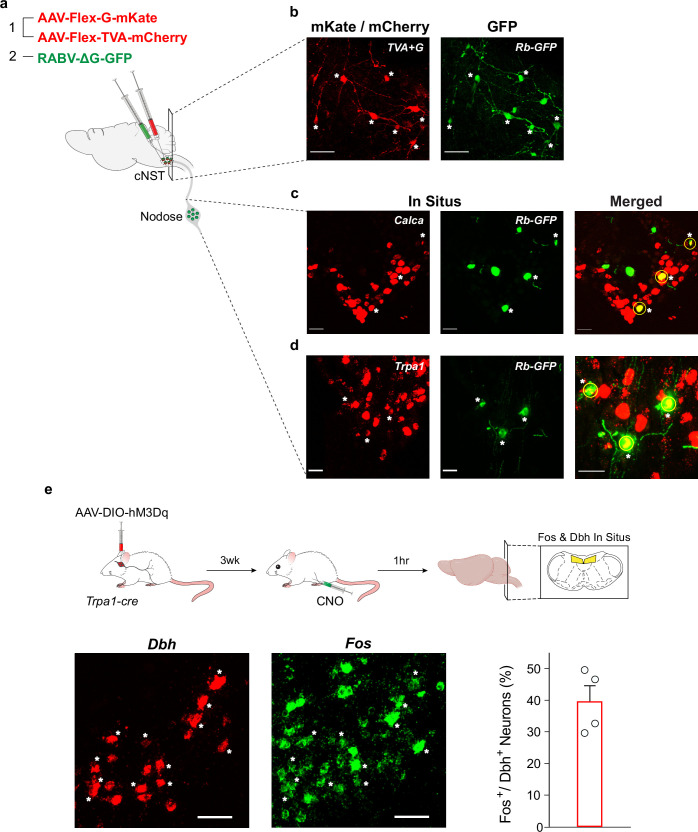
A vagal to cNST circuit. **a**, Strategy for targeting a green fluorescently labelled retrograde transsynaptic rabies reporter (RABV–GFP)^46,47,79^ to the cNST. DBH neurons in the cNST (in *Dbh-cre* animals) were infected with AAV-Flex-G-mKate (red fluorescence) and AAV-Flex-TVA-mCherry (also red fluorescence) viruses^46,47,79^. The targeted expression of the G-protein and the TVA receptor allows monosynaptic transfer and expression of the RABV-GFP retrograde virus. **b**, DBH Neurons in the cNST that were co-infected by the AAV-G and AAV-TVA viruses and by the RABV-GFP retrograde reporter are highlighted with asterisks. **c**, DBH neurons receive monosynaptic input from CALCA vagal neurons. RNA fluorescence in situ hybridization (in situs) marking CALCA neurons (left panel, red) and GFP from the retrograde virus (middle panel, green), demonstrating that CALCA neurons in the nodose ganglion directly project to DBH neurons in the cNST. The CALCA vagal neurons co-labeled with RABV-GFP are indicated by asterisks. The right panel shows the merged view. n = 3 mice, Scale bars, 50 μm. The fraction of Calca neurons that are labeled by the transsynaptic reporter is 12.2 ± 1.4%. **d**, DBH neurons receive monosynaptic input from TRPA1 vagal neurons. RABV-GFP from DBH neurons in the cNST retrogradely labels TRPA1 vagal neurons. The TRPA1 vagal neurons co-labeled with RABV-GFP are indicated by asterisks. The right panel shows an enlarged merged view. n = 3 mice, Scale bars, 50 μm. The fraction of Calca neurons that are labeled by the transsynaptic reporter is 13.1 ± 3.5%. Overall, only a small fraction of the TRPA1 (or Calca) neurons are labeled by the retrograde virus. This is expected due to: (i) the limited efficiency of the TVA retrograde labeling system, (ii) TRPA1 neurons represent multiple functional types, for example those that carry signals informing the brain of intestinal fat versus those that report inflammatory responses^5^ (see Extended Data Fig. 8e), and (iii) DBH neurons would be expected to receive inputs from other vagal neurons that will also be labeled by the retrograde reporter (for instance CALCA and others). **e**, Stimulation of TRPA1 vagal neurons activates DBH neurons in the cNST. An excitatory DREADD receptor (via AAV-DIO-hM3Dq) was targeted bilaterally to the nodose ganglion of *Trpa1-cre* mice. The mice were then examined for the induction of Fos in the cNST. Lower panels show in-situ hybridizations for *Dbh* (red) and *Fos* (green). Scale bars, 50 μm. Approximately 40% of DBH neurons are activated in response to TRPA1 neuron stimulation (bar graph). Schematics in panels **a**,**e** were created using BioRender (https://biorender.com).

To demonstrate that the cNST DBH-expressing neurons receive direct input from the vagal ganglion neurons carrying the anti-inflammatory (expressing TRPA1) and pro-inflammatory (expressing CALCA) signals, we used a Cre-dependent monosynaptic retrograde viral reporter system. In essence, we infected the cNST of *Dbh-cre* animals with an AAV carrying a Cre-dependent glycoprotein coat and a surface receptor for a transsynaptic reporter. We then infected the DBH neurons expressing the viral receptor and G protein with a retrograde rabies reporter (RABV–dsRed)^46,47^, and examined whether they receive input from TRPA1 and CALCA vagal ganglion neurons. The results shown in Extended Data Fig. 12 demonstrate the transfer of the rabies reporter from the cNST to the vagal TRPA1 and CALCA neurons, confirming the monosynaptic connections between the immune-responding neurons in the vagal ganglia and DBH neurons in the cNST. Next, we targeted the excitatory DREADD to TRPA1 vagal neurons and showed that their stimulation indeed robustly activated DBH neurons in the cNST (Extended Data Fig. 12e).

Together, these results uncovered two lines of signalling from the vagal ganglia to the brain. One line (TRPA1) carries anti-inflammatory signals and acts on cNST neurons to enhance the anti-inflammatory response (for example, by positive feedback onto immune cells releasing anti-inflammatory cytokines) and helps to suppress the pro-inflammatory state. The other (CALCA neurons) responds to pro-inflammatory signals and helps to tune down the pro-inflammatory response (for example, by negative feedback onto immune cells releasing pro-inflammatory cytokines).

Activation of other vagal populations did not significantly impact the LPS-induced inflammatory responses (Extended Data Fig. 9), further illustrating the specificity of this body–brain circuit.

## Restoring immune balance

**Fig. 6:**
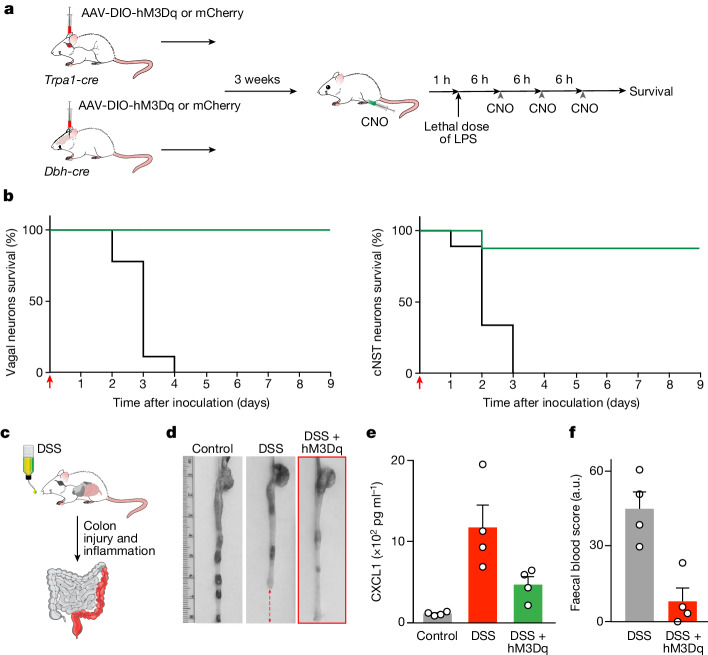
Vagal–brain restoration of immune balance. **a**, Activation of TRPA1 vagal neurons and DBH cNST neurons. AAV-DIO-hM3Dq was targeted bilaterally to the nodose ganglion of *Trpa1-cre* mice or the cNST of *Dbh-cre* mice. Control Cre-driver mice received AAV-DIO-mCherry. Mice were challenged with a lethal dose of LPS (see Methods), and the TRPA1 vagal or the DBH cNST neurons were activated by injection of CNO at 6-h intervals beginning 1 h before injection of LPS (four injections over 19 h). **b**, Activation of TRPA1 vagal (left) or DBH cNST (right) neurons rescues animals from LPS-induced sepsis. The graphs show survival curves. All groups received the same regime of CNO injections. mCherry (black lines; *n* = 9) and hM3Dq (green lines; *n* = 8 (vagal) and *n* = 9 (cNST)) are shown. Log-rank (Mantel–Cox) tests: *P* < 0.001 (vagal) and *P* < 0.001 (cNST). The red arrow denotes LPS injection. All mCherry control mice, in both groups, died within the first 4 days. **c**, DSS-induced ulcerative colitis. **d**, Activation of TRPA1 vagal neurons protects animals from DSS-induced colon damage. AAV-DIO-hM3Dq or mCherry was targeted bilaterally to the nodose ganglion of *Trpa1-cre* mice. All animals were provided with CNO in the drinking water (see Methods). Note the effect of DSS-induced inflammation on colon integrity (middle); the red arrows illustrate the loss of the distal colon in DSS-treated animals, but not in DSS-treated animals if this circuit was activated (right; *n* = 4; similar protection was observed in all animals). **e**, Bar graphs show levels of CXCL1 pro-inflammatory cytokine in control, DSS-treated and DSS-treated in combination with activation of TRPA1 vagal neurons. Values are mean ± s.e.m.; Mann–Whitney *U*-test: *P* = 0.03 (DSS, DSS + hM3Dq). **f**, Significant levels of occult stool blood is detected in the DSS-treated but not in the TRPA1 neuron-activated animals. Values are mean ± s.e.m.; Mann–Whitney *U*-test: *P* = 0.03. Schematics in panels **a**,**c** were created using BioRender (https://biorender.com).

We reasoned that exogenous activation of the body–brain anti-inflammatory regulatory circuit should protect animals from a runaway inflammatory response. Therefore, we injected control mice with lethal doses of LPS^48^ (that is, overwhelming the natural innate response) and performed the same injections in animals where this circuit had been chemogenetically activated by targeted expression of excitatory DREADD to the TRPA1 vagal neurons (Fig. 6a). In parallel, we also targeted the DBH-expressing neurons in the cNST. Remarkably, chemogenetic activation of either of these neuronal populations in this immunomodulatory circuit is sufficient to dramatically transform the survival of these animals to an otherwise lethal dose of LPS: approximately 90% of the mice were alive after the immune challenge (Fig. 6b).

Next, we used a mouse model of ulcerative colitis (dextran sodium sulfate (DSS)-induced intestinal inflammation)^49^ to examine whether activation of this immunomodulatory circuit can prevent the dramatic loss of colon integrity, increase of pro-inflammatory cytokine levels and high levels of faecal blood observed in this model of colon injury and inflammation. We exposed control mice and animals in which the TRPA1 vagal neurons had been chemogenetically activated by targeted expression of excitatory DREADD to DSS for 7 days (see Methods for details); this time is sufficient for the development of the severe pathologies triggered by DSS treatment^50^. DSS-treated control animals exhibited dramatic damage to the distal colon, showed significant occult stool blood and had a major increase in the levels of pro-inflammatory cytokines (Fig. 6c–f). By contrast, chemogenetic activation of the TRPA1 vagal neurons protected animals from all three pathological conditions (Fig. 6c–f, hM3Dq animals).

**Extended Data Fig. 13:**
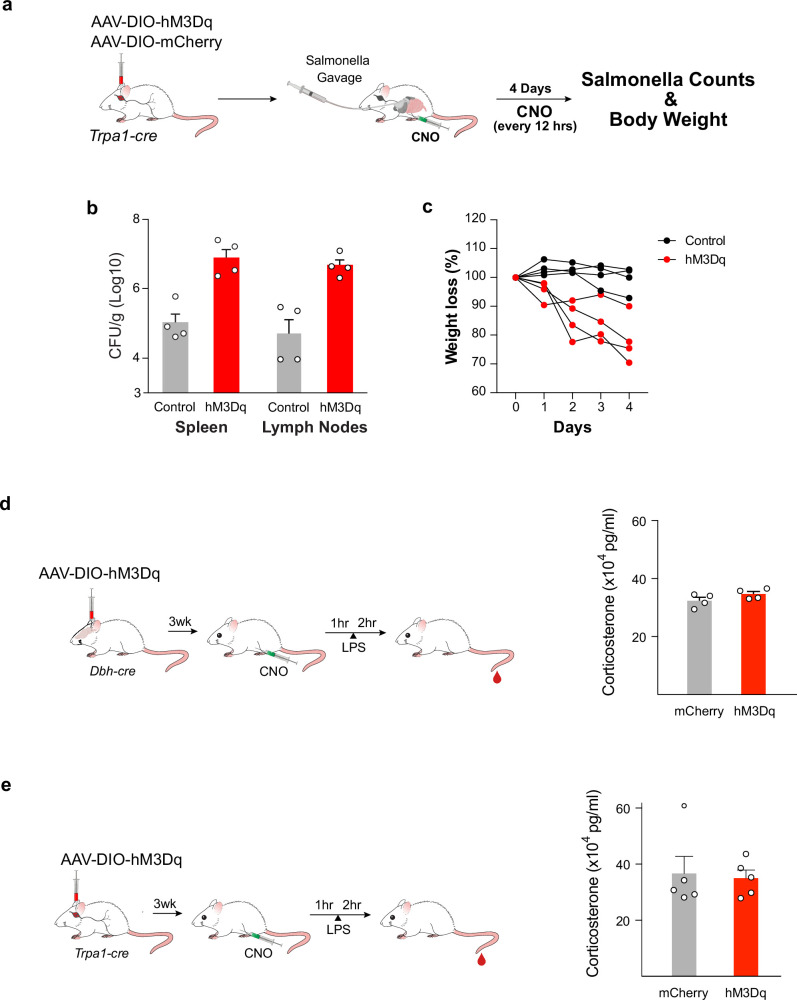
Activation of TRPA1 vagal neurons impacts the course of Salmonella infection. We reasoned that strong and sustained activation of the TRPA1 vagal neurons would drive a potent anti-inflammatory state, and this would be expressed as heightened susceptibility to bacterial infection. To test this prediction, we infected mice by gut gavage with *Salmonella enterica* Serovar Typhimurium (STm)^51^, and monitored the course of infection over 5 days. **a**, Strategy for chemogenetic activation of TRPA1 vagal neurons. An excitatory DREADD receptor (via AAV-DIO-hM3Dq) was targeted bilaterally to the nodose ganglion of *Trpa1-cre* mice. Control mice received injections of AAV-DIO-mCherry. Mice were infected with STm (1~2 x10^7 CFU) via oral gavage. We maximally activated the circuit by injecting CNO at 12 h intervals for a total of 8 injection beginning 12 h prior to STm infection. Body weight was monitored daily. **b**, Activation of TRPA1 vagal neurons impairs protection against STm infeciton. Left graph shows the load of STm in the spleens and mesenteric lymph nodes (LNs) of mice expressing the excitatory DREADD (hM3Dq; n = 4 mice) or control reporter (mCherry; n = 4 mice). Note the nearly 2-log increase in STm load in the spleen and LN of TRPA1-activated animals, reflecting the suppressed pro-inflammatory state. Values are means ± SEM; Mann–Whitney U-test, spleen, p = 0.03; LN, p = 0.03. **c**, As expected, these animals also experienced a severe loss of body weight during the course of STm infection (right panel). **d**, Activation of the vagal-brain axis does not alter the levels of circulating corticosterone induced by LPS. An AAV virus carrying the hM3Dq excitatory DREADD, was targeted to the cNST of *Dbh-cre* mice for chemogenetic activation. Control animals received an injection of AAV-DIO-mCherry. The bar graphs show levels of corticosterone in the peripheral blood of mice expressing the excitatory DREADD (hM3Dq; n = 4 mice) or control reporter (mCherry; n = 4 mice). Blood samples were collected 2 h after LPS stimulation, and all animals were given CNO 1 hr prior to LPS injection. Values are means ± SEM; Mann–Whitney U-test, p = 0.32. **e**, An AAV virus carrying the hM3Dq excitatory DREADD, was targeted to the TRPA1 vagal neurons for chemogenetic activation. The bar graphs show the levels of corticosterone in the peripheral blood of the mice expressing the excitatory DREADD (hM3Dq; n = 5 mice) or control reporter (mCherry; n = 5 mice). Values are means ± SEM; Mann–Whitney U-tests, p = 0.84. Schematics in panels **a**,**d**,**e** were created using BioRender (https://biorender.com).

Finally, we hypothesized that activation of this body–brain immune regulatory circuit should influence responses to infection models. We reasoned that a persistent and artificially strong activation should lead to a severely reduced pro-inflammatory state and suppressed innate immune responses. To test this, we established a model of bacterial infection using intestinal *Salmonella enterica* subspecies *enterica* serovar Typhimurium^51^. As predicted (Extended Data Fig. 13a–c), strong, sustained activation of this circuit resulted in a dramatic increase in bacterial load in the DREADD-activated hosts, but not in control infected animals.

## Discussion

A well-controlled innate immune response is the key to fighting an immune insult, while minimizing the risk of a dangerous out-of-control pro-inflammatory reaction. The brain has long been proposed to act as a master modulator of body biology, including organ function^1,2^, nutrient preference^5,6^ and metabolism^3^. The involvement of the vagus nerve in sickness (including fever, plasma corticosterone, hyperalgesia, and suppression of social and feeding behaviours)^23^ has long been appreciated. Over 20 years ago, pioneering studies by Tracey and colleagues^18,52^ have demonstrated that broad electrical stimulation of the whole vagal nerve bundle (that is, thousands of random different fibres, including afferent and efferent pathways) can protect animals from shock produced by high levels of TNF. That work helped to reveal the importance of the vagus nerve in modulating levels of TNF and inflammation^52^. More recently, it has been shown that chemical activation of vagal fibres^53^ also reduced the levels of TNF after LPS. However, the nature of the candidate body–brain circuit, the identity and role of the neuronal elements, and the logic of the system remained largely unknown.

Here we showed that cytokines themselves mediate the activation of the vagal–brain axis and characterized the key neuronal elements and the logic of the circuit. Most unexpectedly, this body–brain circuit modulates not only pro-inflammatory^18,52^ but also the anti-inflammatory response. Indeed, we identified a population of vagal neurons that respond to pro-inflammatory signals and a different population responding to anti-inflammatory signals that transfer inflammatory information from the body to neurons in the cNST.

This body–brain circuit monitors the development of an inflammatory response and ensures the homeostatic balance between the pro-inflammatory and anti-inflammatory state. Critically, removing this body–brain circuit during an innate immune challenge abolishes essential immune regulation, and an otherwise normal inflammatory response becomes unregulated and out of control. By contrast, exogenous activation of this circuit during an immune response can reduce the pro-inflammatory state while promoting anti-inflammatory responses. We propose that the cNST neurons function as a biological rheostat controlling the extent of the peripheral inflammatory response by exerting positive-feedback and negative-feedback modulation on immune cells. In this regard, we anticipate that the two vagal lines of information, from the periphery to the brain, will interact at the level of the cNST to coordinate the appropriate descending signals. It will be interesting to determine the nature of the cNST DBH-positive neurons targeted by each vagal line.

Dysregulation of the immune system, and an enhanced pro-inflammatory state, has been linked to a broad range of diseases, from diabetes^54^ to neurodegeneration^55^, attesting to the importance of a proper immune balance. Activation of the DBH-expressing neurons in the cNST during an immune response did not alter the levels of circulating corticosterone induced by LPS (Extended Data Fig. 13d,e). We suggest that pharmacologically targeting this circuit may provide exciting new strategies to modulate and manage immune disorders, including autoimmune diseases (for example, rheumatoid arthritis), cytokine storm, toxic shock and other hyperactive immune states, such as those promoted by new immunotherapies^56^. In the future, it would be of great interest to identify additional neuronal populations that may participate in this process and characterize the elements of this immunomodulatory body–brain circuit, including the nature of other ascending signals, descending signals and effectors.

## Methods

### Animals

All procedures were performed in accordance with the US National Institutes of Health (NIH) guidelines for the care and use of laboratory animals, and were approved by the Columbia University Institutional Animal Care and Use Committee. Both male and female mice at least 7 weeks of age were used in the study. C56BL/6J (JAX 000664); *Myd88*^−/−57^ (JAX 009088); TRAP2 (ref. ^35^) (JAX 030323); *Dbh-cre*^38^ (JAX 033951); *Vip-IRES-cre*^58^ (JAX 010908); *Gpr65-IRES-cre*^59^ (JAX 029282); *Piezo2-cre*^60^ (JAX 027719); *Oxtr-IRES-cre*^61^ (JAX 030543); *Vglut2-IRES-cre*^62^ (JAX 028863); *Vgat-IRES-cre*^62^ (JAX 016962); Ai9 (ref. ^63^) (JAX 007909); Ai96 (ref. ^64^) (JAX 028866); Ai162 (ref. ^65^) (JAX 031562); and Rosa-iDTR^66^ (JAX 007900) were obtained from the Jackson Laboratory. *Trpa1-IRES-cre*^5^ was generated in the Zuker laboratory. *Calca-cre*^45^ mice were a gift of R. Palmiter.

### FOS stimulation and histology

Mice housed in their home cages were injected intraperitoneally with LPS (50 μg kg^−1^; 14011, Cell Signaling Technology), lipoteichoic acid (LTA; 1 mg kg^−1^; L2512, Sigma), flagellin (20 μg kg^−1^; SRP8029, Sigma Aldrich), profilin (20 μg kg^−1^; SRP8050, Sigma Aldrich), Zymosan (2.5 mg kg^−1^; Z4250, Sigma Aldrich), IL-10 (100 μg kg^−1^; 575804, BioLegend), a cocktail of IL-6 (100 μg kg^−1^; 575706, BioLegend), IL-1β (100 μg kg^−1^; 401-ML, R&D) and TNF (100 μg kg^−1^; 410-MT, R&D) or saline control (0.9% NaCl). Then, 2 h later, these mice were perfused transcardially with PBS followed by 4% paraformaldehyde. Brains were dissected, fixed in 4% paraformaldehyde overnight at 4 °C and then sliced coronally at 100 μm thickness. The brain sections were permeabilized and blocked with 10% normal donkey serum (S30, EMD Millipore) in PBS containing 0.3% Triton X-100. Sections were incubated with an anti-FOS primary antibody (226004, SYSY; guinea pig, diluted 1:5,000) at 4 °C overnight, followed by labelling with a secondary antibody (Alexa Fluor 647-conjugated donkey anti-guinea pig; 706605148, Jackson ImmunoResearch) at room temperature for 2 h. For RNA in situ hybridizations, fixed-frozen nodose ganglia or brains were sectioned at 16 μm thickness and processed for mRNA detection using the RNAscope Fluorescent Multiplex Kit (Advanced Cell Diagnostics) following the manufacturer’s instructions. The following RNAscope probes were used: *Fos* (316921-C2), *Dbh* (464621-C1), *Trpa1* (400211-C3), *Calca* (578771-C2) and GFP (400281-C1). Images were acquired using an Olympus FluoView 1000 confocal microscope. Quantification of fluorescent signals was carried out by manually counting the number of positive neurons.

### Stereotaxic surgery

All stereotaxic surgery procedures were carried out using aseptic technique. Mice were anaesthetized with a mixture of ketamine and xylazine (100/10 mg kg^−1^, intraperitoneally) and then positioned on a custom-built stereotaxic frame equipped with a closed-loop heating system to maintain their body temperature. The viral constructs were injected into the cNST through a small craniotomy. The injection coordinates (based on Paxinos stereotaxic coordinates) for virus delivery in the cNST were as follows: caudal 7.5 mm, lateral ±0.3 mm and ventral 3.7–4 mm, all relative to Bregma and the skull surface. In chemogenetic experiments, TRAP2, *Dbh-cre*, *Vglut2-cre* and *Vgat-cre* mice received bilateral injections of 200 nl of AAV9-Syn-DIO-hM3Dq (#44361-AAV9, Addgene) and 300 nl of AAV9-Syn-DIO-hM4Di (#44362-AAV9, Addgene) in the cNST. Equivalent volumes of AAV9-Syn-DIO-mCherry (#50459-AAV9, Addgene) were injected as controls. For fibre photometry experiments, *Vglut2-cre* mice were unilaterally injected with 100 nl of AAV9-Syn-Flex-GCaMP6s (#100845-AAV9, Addgene) in the cNST, and an optical fibre (400-μm core, 0.48 NA, Doric Lenses) was implanted 50–100 μm above the GCaMP virus injection site.

### Fibre photometry and subdiaphragmatic vagotomy

Photometry experiments were conducted at least 14 days after the stereotaxic viral injection and fibre implantation (see the section on stereotaxic surgery for details). Before the experiments, mice were acclimated to the recording chamber for 1 h per day over 3 consecutive days. On the fourth and fifth days, mice were recorded for the bulk GCaMP responses to saline and LPS (0.5 mg kg^−1^), respectively, in a 5-h recording session. Saline and LPS were intraperitoneally injected 15 min after the onset of recording. Real-time population-level GCaMP fluorescence signals were detected, amplified and recorded using a RZ5P fibre photometry system with Synapse software (Tucker Davis Technologies), as previously described^6,67^. The collected data were downsampled, detrended and smoothed by a custom MATLAB code. The calcium transients were identified as previously described^68–70^, and the area under the curve (AUC) was calculated by integrating the fluorescence signal under identified calcium transients.

To assess the necessity of the vagus nerve in the cNST responses to LPS, a separate group of *Vglut2-cre* mice received bilateral subdiaphragmatic vagotomy as previously described^5,34^, following the injection of GCaMP virus and the implantation of the fibre in the cNST. Mice were anaesthetized with ketamine and xylazine (100/10 mg kg^−1^, intraperitoneally). The stomach and oesophagus were carefully exposed to avoid any damage to blood vessels or the liver. The dorsal and ventral branches of the vagus nerve along the subdiaphragmatic oesophagus were then exposed, and the right and left vagus nerves were transected. The abdominal muscle layer and skin were closed with sutures. Following the vagotomy procedure, the mice were given 2 weeks to recover before fibre photometry recordings. The expression of GCAMP and placement of optic fibres were histologically verified at the termination of the experiments.

### Genetic access to LPS-activated neurons in the brain

The TRAP^35^ strategy was used in TRAP2 mice to gain genetic access to LPS-activated neurons in the cNST. The AAV-injected TRAP2 mice (2–3 weeks after viral injection) or TRAP2;Ai9 mice were first habituated to intraperitoneal injections by daily injection of 100 μl saline for 5 days. After habituation, LPS (50 μg kg^–1^) was given intraperitoneally, then 90 min later, 4-hydroxytamoxifen (4-OHT; 20 mg kg^−1^; H6278, Sigma) was administered. Mice were used for experiments a minimum of 4 weeks after this TRAP protocol; this extended waiting time is crucial to restore sensitivity to LPS after the initial LPS-induced TRAPing^71^.

### Chemogenetic manipulation experiments and measurement of cytokines

Following bilateral injection with AAV9-Syn-DIO-hM3Dq in the cNST of *Dbh-cre*, *Vglut2-cre* or *Vgat-cre* mice, or in the nodose ganglion of *Trpa1-cre*, *Calca-cre*, *Vip-cre*, *Gpr65-cre*, *Piezo2-cre* and *Oxtr-cre* mice, the animals were allowed to recover for a minimum of 3 weeks before treatment with CNO (BML-NS105, Enzo Life Sciences). When using TRAP2 animals, at least 4 weeks elapsed between TRAPing and CNO treatment. Two doses (2 mg kg^−1^ and 1 mg kg^−1^) of CNO were given intraperitoneally at 12 h and 1 h before saline or LPS stimulation. Two hours after the intraperitoneal injection of saline or LPS (0.1 mg kg^−1^), blood samples were collected from either the submandibular or the tail vein. Cytokines in the blood were measured using commercially available ELISA kits (R&D), following the manufacturer’s instructions. Saline and LPS experiments were conducted on the same cohort of mice but at least 7 days apart.

To examine LPS-induced cytokine responses over time, wild-type (C56BL/6J) mice were injected intraperitoneally with saline or LPS, and peripheral blood samples were collected at 0, 2, 4 and 6 h post-stimulation.

To measure circulating cytokine levels following administration of exogenous cytokines, mice were injected intraperitoneally with IL-6 (100 μg kg^−1^), TNF (100 μg kg^−1^) or IL-10 (100 μg kg^−1^), and peripheral blood samples were harvested at 10 min and 2 h post-injection.

To ‘clamp’ pro-inflammatory cytokine levels, a cocktail of IL-6 (300 μg kg^−1^), IL-1β (15 μg kg^−1^) and TNF (30 μg kg^−1^) was injected with LPS.

### Saporin ablation of DBH cNST neurons

Previous studies have shown that saporin-mediated targeted ablation is a highly effective method to kill DBH-expressing neurons^72,73^. We bilaterally injected the cNST of mice with an anti-DBH–saporin conjugate (20 ng per side; IT-03, Advaced Targeting Systems), and after 2–3 weeks recovery, animals were stimulated with LPS (0.1 mg kg^−1^) intraperitoneally. One hour following the LPS injections, blood samples were collected for measuring cytokine levels in the control and anti-DBH–saporin-treated animals.

### scRNA-seq of cNST and LPS-TRAPed cells

To perform scRNA-seq^74,75^ on the entire cNST, we isolated single cells from the cNST as previously described^76^ with the following modifications. In brief, mice were anaesthetized with isoflurane and transcardially perfused with ice-cold carbogenated (95% O_2_ and 5% CO_2_) NMDG-HEPES-ACSF (93 mM NMDG, 2.5 mM KCl, 1.2 mM NaH_2_PO_4_, 30 mM NaHCO_3_, 20 mM HEPES, 25 mM glucose, 10 mM MgSO_4_, 1 mM CaCl_2_, 1 mM kynurenic-acid Na salt, 5 mM Na-ascorbate, 2 mM thiourea and 3 mM Na-pyruvate, pH 7.4). The brainstems were rapidly extracted and sliced into 300-μm sections containing the cNST using a vibratome (VT-1000S, Leica) in ice-cold NMDG-HEPES-ACSF solution with continuous carbogenation. The cNSTs were dissected, pooled (from five animals) and digested in Trehalose-HEPES-ACSF (92 mM NaCl, 2.5 mM KCl, 1.25 mM NaH_2_PO_4_, 30 mM NaHCO_3_, 20 mM HEPES, 25 mM glucose, 2 mM MgSO_4_, 2 mM CaCl_2_, 1 mM kynurenic-acid Na salt and 2.5 wt/vol trehalose, pH 7.4) containing papain (20 U ml^−1^; LK003150, Worthington) and DNase I (25 U ml^−1^) at 35 °C for approximately 1 h. Using Pasteur pipettes with progressively narrowing tip diametres, the tissue was triturated in DNase I-containing (25 U ml^−1^) Trehalose-HEPES-ACSF solution to form single-cell suspension. The dissociated cells were passed through a 40-μm filter and resuspended in resuspension-ACSF (117 mM NaCl, 2.5 mM KCl, 1.25 mM NaH_2_PO_4_, 30 mM NaHCO_3_, 20 mM HEPES, 25 mM glucose, 1 mM MgSO_4_, 2 mM CaCl_2_, 1 mM kynurenic-acid Na salt and 0.05% BSA, pH 7.4). The resulting cell suspension was processed by the Columbia Genome Core to encapsulate and barcode individual cells using the 10X Genomics Chromium system.

For sequencing LPS-TRAPed cells, we used TRAP2;Ai9 mice that were TRAP-labelled with tdTomato in response to LPS. Cells from the cNST were isolated as described above. The cell suspension was stained with DRAQ5 (62254, Thermo Scientific) and Calcein Violet (C34858, Thermo Scientific) to label viable cells, before FACS. A total of 288 tdTomato-positive LPS-TRAPped cells and 96 tdTomato-negative cells were sorted into 96-well plates pre-loaded with cell lysis buffer containing 0.1% Triton X-100, 0.5 U ml^–1^ SuperaseIN (AM2694, Ambion), 1 mM dNTP and 1 μM capture primer (that is, barcoding). cDNA was synthesized using Maxima Reverse Transcriptase (EP0753, Thermo Scientific) according to the manufacturer’s instruction. cDNA from all the wells/cells was combined, followed by clean-up using Silane beads (37002D, Thermo Scientific). Pooled cDNA was amplified using Kapa HotStart Mix with SMART PCR primer (0.2 μM), and then purified using AMPureXP beads (A63880, Beckman Coulter Life Sciences). Of cDNA, 0.6 ng was used as input to prepare libraries using Nextera XT kit (FC-131-1024, Illumina). The resulting libraries were sequenced on an Illumina sequencer.

### scRNA-seq data analysis

Illumina sequencing reads were mapped to the mouse genome using the CellRanger pipeline with the default parameters. Analysis of scRNA-seq data, including the generation of cell clusters and identification of neuronal cluster markers, was performed using a custom R code developed following Seurat online instructions and vignettes^77,78^. We removed genes that were expressed in fewer than ten cells in the cNST-seq dataset, and in fewer than three cells in the TRAP2-seq dataset. In addition, we removed cells with low-depth sequencing (fewer than 2,000 genes in the cNST-seq dataset). To integrate datasets from cNST-seq and TRAP2-seq, we used the standard scRNA-seq integration procedure as outlined by Seurat (https://satijalab.org/seurat/). In brief, we first normalized each dataset and then used the Seurat ‘FindVariableGenes’ routine to identify 2,000 variable genes from each sample. Then, a common set of variable features were determined by Seurat ‘SelectIntegrationFeatures’ to merge samples. Finally, the first 25 principal components were used for generating cell types utilizing Seurat ‘FindClusters’.

### Nodose ganglion injection experiments

The injection of AAV to nodose ganglion was performed as previously described^5^. In brief, Cre-expressing mice (*Trpa1-cre*, *Calca-cre, Vip-cre*, *Piezo2-cre*, *Gpr65-cre* and *Oxtr-cre*) were anaesthetized with intraperitoneal administration of ketamine and xylazine (100/10 mg kg^−1^). The skin under the neck was shaved and an incision (approximately 1.5 cm) in the midline was made. The trachea and surrounding muscles were gently retracted to expose the nodose ganglia. A mixture of Fast Green (F7252, Sigma) and AAV carrying the Cre-dependent excitatory DREADD (AAV9-Syn-DIO-hM3Dq) or control (AAV9-Syn-DIO-mCherry) was injected to both left and right ganglia using a 30° beveled glass pipette (Clunbury Scientific). The injection volume per ganglion was 300 nl. For experiments ablating TRPA1 neurons, we crossed *Tpra1-cre* mice to Rosa-DTR mice^66^, and bilaterally injected vagal ganglia with control PBS alone or PBS containing 2 ng DTX (200 nl total volume; D0564, Sigma Aldrich)^44^. At the end of surgery, the skin incision was closed using 5-0 absorbable sutures (421A, CP Medical). Following the procedure, mice were allowed to recover for a minimum of 21 days before testing. The viral expression and ablation efficiency were histologically confirmed by examining the nodose ganglia extracted from all tested animals; mice with insufficient viral expression, mistargeting of viral injection or unsuccessful ablation were removed from data analysis.

### Vagal calcium imaging

Calcium imaging of the nodose ganglion was conducted as previously described^5,6^. For imaging in response to intragastric delivery of glucose (or linoleic acid) and intraperitoneal injections of saline control and cytokines, a typical recording session consisted of: (1) saline; (2) one of the three pro-inflammatory cytokines (TNF, IL-1β or IL-6); (3) anti-inflammatory cytokine (IL-10); and (4) and (5) two trials with glucose (or linoleic acid). Each trial was 5 min. Cytokines were injected 1 min after the onset of the recording. Glucose (500 mM) and linoleic acid (10%) was delivered intragastrically as previously described^5,6^. For all experiments, we used 100 μg kg^−1^ of each cytokine. To deliver cytokines extraintestinally, we placed a segment of the intestine in a custom-made perfusion chamber while still keeping it connected to the remainder of the gastrointestinal tract (no carbogenation). Each recording session included six interleaved trials, with two trials for each stimulus. Trials were 15 min long, and consisted of a 180-s baseline (saline), a 180-s cytokine or control stimulus and a 9-min washout (saline) period. The flow rates were maintained at around 600 μl min^−1^ throughout the experiment to minimize mechanical responses that may occur during the transition between trials. Cytokines were dissolved in saline at the concentration of 1 μg ml^−1^. During the entire perfusion session, all of the solutions were maintained at 37 °C.

### Calcium imaging data collection and analysis

Imaging data were acquired exactly as previously described^5,6^. Neuronal activity was analysed for significant stimulus-evoked responses as described in ref. ^6^. We first computed the baseline distribution of deviations from the median for each cell throughout the entire experiment using periods before the stimulus delivery. Subsequently, this baseline was utilized to derive a modified *z*-score by subtracting the median and dividing by the median absolute deviation. Trials with an average modified *z*-score above 1.6 for the 180 s (stimuli delivered via intraperitoneal) or 480 s (stimuli delivered via perfusion) following the initiation of stimulation were classified as responding trials (all responders had minimal peak amplitudes of 1% Δ*F*/*F)*. *z*-Scores from responders were normalized across stimuli to generate heatmaps of normalized fluorescence traces (see also refs. ^5,6^).

### Mapping vagal-to-cNST circuit

For monosynaptic retrograde tracing experiments, the cNST of *Dbh-cre* animals were first injected with a 1:1 mixture of AAV1-DIO-TVA-mCherry and AAV1-DIO-G(N2C)-mKate^46,47,79^ followed by a second injection of EnvA-pseudotyped G-deleted rabies virus carrying a GFP reporter (RABV-N2C(ΔG)-GFP-EnvA)^46,47,79^ 3 weeks later. Seven to ten days after RABV infection, the animals were euthanized to identify and examine presynaptic neurons in the nodose ganglion by RNA in situ hybridization.

To determine whether DBH neurons are activated by stimulation of TRPA1 vagal neurons, AAVs carrying the Cre-dependent excitatory DREADD (AAV9-Syn-DIO-hM3Dq) were injected into the nodose ganglia of *Trpa1-cre* mice (see the section ‘Nodose ganglion injection experiments’). Following injection, the animals were allowed to recover for a minimum of 3 weeks before TRPA1 vagal neuron activation with CNO. CNO (5 mg kg^−1^) was injected intraperitoneally, and 1 h later, mice were euthanized to examine co-expression of *Fos* and *Dbh* in the cNST by in situ hybridization.

### Modulation of survival in LPS-induced endotoxaemia through chemogenetic activation of the vagal–brainstem axis

After bilateral injection of AAV9-Syn-DIO-hM3Dq or AAV9-Syn-DIO-mCherry (control) in the cNST of *Dbh-cre* mice and in the nodose ganglion of *Trpa1-cre* mice, animals were allowed to recover for a minimum of 3 weeks before the injection of LPS. CNO (5 mg kg^−1^) was intraperitoneally administered 1 h before a lethal dose of LPS (12.5 mg kg^−1^)^48^. Following the LPS challenge, CNO (5 mg kg^−1^) was administered every 6 h for a total of three doses; survival was monitored every 6 h.

### DSS-induced colitis and chemogenetic activation of TRPA1 vagal neurons

*Trpa1-cre* mice were injected bilaterally in the nodose ganglia with AAV9-Syn-DIO-hM3Dq or control AAV9-Syn-DIO-mCherry. Three weeks later, they were exposed to 3% DSS in the drinking water^50^ for 7 days. CNO (0.03 mg ml^−1^) was added to DSS solution of the experimental cohort to concomitantly activate TRPA1 neurons. To motivate mice to drink, 10 mM Acek was added to the drinking mix in both groups. Colon morphology was examined at the termination of the experiment; CXCL1 levels were measured using ELISA (R&D). Faecal occult blood was monitored using Hemoccult Dispensapak Plus (61130, Beckman Coulter) according to the manufacturer’s instructions.

### *S. enterica* serovar Typhimurium infection and chemogenetic activation of TRPA1 vagal neurons

*Trpa1-cre* mice injected with AAV9-Syn-DIO-hM3Dq or AAV9-Syn-DIO-mCherry (control) in the nodose were allowed 3–4 weeks for virus reporter expression, and then infected with 1–2 × 10^7^ colony-forming units of *S. enterica* serovar *Typhimurium* (14028, American Type Culture Collection) through oral gavage^51^. CNO (5 mg kg^−1^) was injected at 12-h intervals beginning 12 h before *S. enterica* serovar *Typhimurium* gavage, for a total of eight injections over 4 days. As a proxy for the health of the animal, we monitored body weight daily. At day 5 post-infection, the tissues (spleen and mesenteric lymph nodes) were collected from the infected mice, homogenized for serial dilutions in PBS and plated on LB agar^51^; colony-forming units were counted after overnight incubation of the plates at 37 °C.

### Statistics

No statistical methods were used to predetermine sample size, and investigators were not blinded to group allocation. No method of randomization was used to determine how animals were allocated to experimental groups. Statistical methods used include Mann–Whitney *U*-test, Wilcoxon test, one-way analysis of variance and log-rank (Mantel–Cox) test, and are indicated for all figures. All of the statistical tests are two-tailed. Analyses were performed in MATLAB, R, Python and GraphPad Prism 8. Data are presented as mean ± s.e.m.

### Inclusion and ethics

We support an all-inclusive, diverse and equitable conduct of research.

### Reporting summary

Further information on research design is available in the Nature Portfolio Reporting Summary linked to this article.

## Online content

Any methods, additional references, Nature Portfolio reporting summaries, source data, extended data, supplementary information, acknowledgements, peer review information; details of author contributions and competing interests; and statements of data and code availability are available at 10.1038/s41586-024-07469-y.

### Supplementary information

Reporting Summary

## Data Availability

All data supporting the findings of this study are available from the corresponding authors (C.S.Z. and H.J.).

## Code availability

The custom code used in this study is available from the corresponding authors (C.S.Z. and H.J.).
